# Hydrolysis of Antimicrobial Peptides by Extracellular Peptidases in Wastewater

**DOI:** 10.1021/acs.est.3c06506

**Published:** 2023-12-16

**Authors:** Natalie Wichmann, Richard Gruseck, Michael Zumstein

**Affiliations:** † Division of Environmental Geosciences, Centre for Microbiology and Environmental Systems Science, University of Vienna, Josef-Holaubek-Platz 2, Vienna 1090, Austria; ‡ Department of Environmental Microbiology, Swiss Federal Institute of Aquatic Science and Technology (Eawag), Überlandstrasse 133, Dübendorf 8600, Switzerland

**Keywords:** antimicrobial peptides, biotransformation, extracellular enzymes, wastewater treatment, LC-HRMS

## Abstract

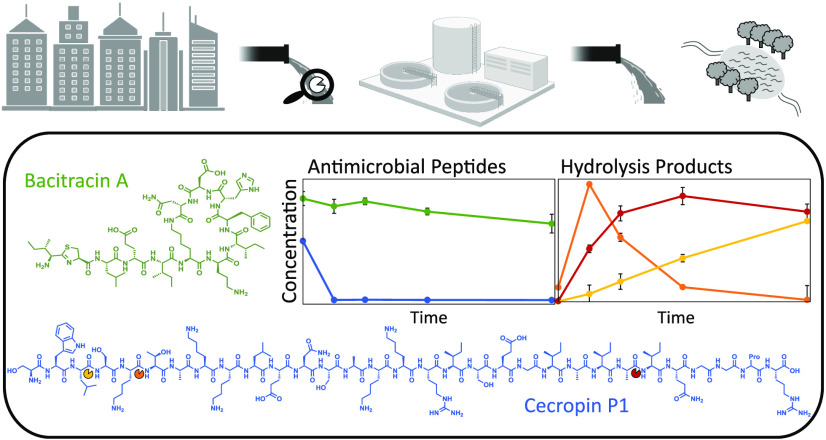

Several antimicrobial peptides (AMPs) are emerging as promising novel antibiotics. When released into wastewater streams after use, AMPs might be hydrolyzed and inactivated by wastewater peptidases—resulting in a reduced release of active antimicrobials into wastewater-receiving environments. A key step towards a better understanding of the fate of AMPs in wastewater systems is to investigate the activity and specificity of wastewater peptidases. Here, we quantified peptidase activity in extracellular extracts from different stages throughout the wastewater treatment process. For all four tested municipal wastewater treatment plants, we detected highest activity in raw wastewater. Complementarily, we assessed the potential of enzymes in raw wastewater extracts to biotransform 10 selected AMPs. We found large variations in the susceptibility of AMPs to enzymatic transformation, indicating substantial substrate specificity of extracted enzymes. To obtain insights into peptidase specificities, we searched for hydrolysis products of rapidly biotransformed AMPs and quantified selected products using synthetic standards. We found that hydrolysis occurred at specific sites and that these sites were remarkably conserved across the four tested wastewaters. Together, these findings provide insights into the fate of AMPs in wastewater systems and can inform the selection and design of peptide-based antibiotics that are hydrolyzable by wastewater peptidases.

## Abstract 2

Investigating extracellular peptidases across wastewater treatment processes, we found highest peptidase activity in influent samples. The kinetics and pathways of antimicrobial peptide hydrolysis by these peptidases were conserved across the tested wastewaters.

## Introduction

The global antibiotic resistance crisis demands the development of new antimicrobial compounds to treat infections caused by pathogenic bacteria.^1,2^ In recent years, several antimicrobial peptides (AMPs) have emerged as promising candidates for novel antibiotics—including AMPs discovered in nature, redesigned from natural structures (i.e., semisynthetic), and rationally designed.^3−9^ Highlighting the promise of AMPs, a recent study showed that some AMPs (i.e., cecropin P1 and R8) caused substantially slower resistance evolution of relevant bacterial strains compared to commonly used small-molecule antibiotics.^10^ Cecropin P1 originates from the parasitic nematode *Ascaris suum* and shows bactericidal effects on a variety of Gram-positive and Gram-negative strains,^11^ while R8 was designed using a linguistic model based on amino acid sequences of known peptides and is active against several clinically relevant bacteria.^7^ Other studies highlighted the promise of the peptidomimetic murepavadin and its derivatives to combat Gram-negative ESKAPE pathogens.^9,12^ Besides promising candidates, several AMPs are already in use. For example, bacitracin is used in veterinary medicine and livestock farming.^13,14^ In human medicine, the AMP daptomycin is widely applied and its use has increased—for example, by 93% during the past decade in Swiss hospitals.^15^

After use, a substantial fraction of human-administered antibiotics enters wastewater streams. AMP administration can occur topically,^3,16^ leading to AMP wash-off and entry into wastewater streams, or systemically.^17^ In previous studies, daptomycin has been detected in urine of patients,^18^ and vancomycin in untreated wastewater.^19,20^ If no complete inactivation occurs during the wastewater treatment process, antibiotics are released into natural environments.^21−25^ The presence of antibiotics in both natural and engineered systems can have effects on the respective microbial communities, may negatively affect ecosystem functions provided by these communities, and can contribute to the emergence and spread of antibiotic resistance.^26−28^ To assess the risks of antibiotics after their use, it is important to understand their fate in wastewater systems—also with respect to transformation by enzymes present in these systems.^29−32^ Given the size and charge density of most AMPs and the resulting impeded cellular uptake, we expect extracellular peptidases to play a key role in the biotransformation of AMPs in wastewater.

A systematic investigation of wastewater peptidases and their potential to hydrolyze AMPs—or other peptide-based chemicals that enter wastewater streams—has not been conducted. In a previous study on the biotransformation of amide-containing antibiotics by enzymes extracted from aeration tanks of municipal wastewater treatment plants (WWTPs), it has been shown that the hydrolysis of the antimicrobial lipopeptide daptomycin can be catalyzed by enzymes extracted from the extracellular polymeric substance (EPS).^33^ In a related study, the specificity of dissolved extracellular peptidases derived from aeration tanks of WWTPs was assessed with a set of model peptides.^34^ The relatively small number of detected hydrolytic events and the high similarity of hydrolyzed amino acid sequences in samples from the three investigated WWTPs suggested that extracellular peptidases in aeration tanks of WWTPs have a considerate substrate specificity, which was partially conserved across WWTPs. Those two studies,^33,34^ as well as the vast majority of work on the biotransformation of anthropogenic organic chemicals in wastewater systems, focused on bioreactors of WWTPs.^35,36^ However, a recent study reported a considerate activity of peptidases in WWTP influent samples (i.e., raw wastewater)—indicating that the role of peptidases present in raw wastewater needs to be considered when studying the fate of peptide-containing chemicals entering wastewater streams.^37^

The objective of this study was to assess the activity of extracellular wastewater peptidases and their potential to hydrolyze AMPs. Therefore, we compared extracellular peptidase activity—measured with a fluorogenic protein probe-based assay—across extracts from five wastewater treatment steps and four WWTPs to identify treatment steps with high activity and thus high potential for enzymatic AMP hydrolysis. We then incubated 10 selected AMPs with the wastewater extracts that showed the highest peptidase activity and investigated the kinetics of AMP biotransformation using liquid chromatography coupled to high-resolution mass spectrometry (HPLC–HRMS). For rapidly removed AMPs, we predicted and searched for products resulting from peptide bond hydrolysis to obtain insights into the hydrolysis pathways of AMPs by wastewater peptidases. For hydrolysis products that were formed by peptidases from all investigated WWTPs and whose concentrations increased during the incubation experiments, we used synthetic peptide standards for absolute quantification.

## Materials and Methods

### Chemicals and Materials

All solutions were prepared using ultrapure water (ELGA PURELAB Pharma Compliance, 0.075 μS). Acetonitrile (hypergrade for LC–MS LiChrosolve, Merck, ≥99.9%, 100292500) was purchased from VWR. Formic acid (98–100%, 5.43804), melittin (honey-bee venom, ≥85%, M2272), colistin sulfate salt (mixture of colistin A and B, C4461), and bacitracin A (31626) were obtained from Sigma-Aldrich. Cecropin P1 trifluoroacetate salt (4039862) was purchased from Bachem. Daptomycin (≥94%, D4229) was purchased from TCI. Murepavadin, omiganan, R8, and tachyplesin I, as well as selected hydrolysis products, were custom-synthesized by SynPeptide Co. Ltd. with purity ≥95%. Chemical structures of the selected AMPs are provided in Figure S1, and selected hydrolysis products are summarized in Table S1. AMP stock solutions were prepared at 1 mg/mL in ultrapure water containing 5% (v/v) acetonitrile. From these stock solutions, an aqueous solution with 5% (v/v) acetonitrile and 0.1% (v/v) formic acid containing all of the AMPs at 10 mg/L was prepared. The latter solution was further diluted in ultrapure water containing 5% (v/v) acetonitrile and 0.1% (v/v) formic acid to obtain a calibration series covering a concentration range from 0.001 to 1 mg/L (Figure S2).

Safe-Lock tubes (0.5, 1.5, and 2 mL), Protein LoBind tubes (0.5 and 2 mL), and epT.I.P.S (0.1–20 and 2–200 μL) were purchased from Eppendorf. 50–1000 μL Universal blue Tips were purchased from VWR. Standard glass HPLC vials (BA10214, amber, 1.5 mL) and glass inserts (702968, 0.1 mL) were purchased from Bruckner Analysetechnik. QuanRecovery MaxPeak HPS vials (HPS Vials) (186009186, 0.3 mL) and TruView pH Control LCMS Certified glass vials (186005663CV) were purchased from Waters. InfinityLab Poroshell 120 Bonus-RP (2.7 μm, 2.1 × 150 mm, 693768-901) and Eclipse Plus C18 RRHD (1.8 μm, 2.1 × 50 mm, 959757-902) chromatography columns were purchased from Agilent. An XSelect Premier CSH C18 column (2.5 μm, 2.1 × 150 mm) with Guard Column Van Guard XSelect Premier CSH C18 (186009870) was purchased from Waters.

### Wastewater Sampling and Enzyme Extraction

We took samples from four municipal WWTPs in Austria (i.e., WWTP A, B, C, and D). Information on these WWTPs, including capacities, pH values of raw influent and of all enzyme extracts, influent loads and temperatures, and solid and hydraulic retention times, is provided in Tables S2 and S3. Where existing, we sampled the following five stages along the wastewater treatment process: influent, primary clarification tank, high-load and low-load aeration tanks, and effluent. WWTP C had an anaerobic treatment tank prior to the aeration tank, while WWTP D had only one aeration tank. We took 2 × 500 mL grab samples at each stage using a plastic beaker. We transferred the samples into 500 mL glass bottles leaving roughly one-third of the bottle empty, transported them to the lab, and pooled duplicate samples for further processing. Enzyme extraction started within 1 h after sampling. For total suspended solid (TSS) determination, we filtered 20 mL of each sample through a preweighed, dry GF/F Whatman glass microfiber filter (Sigma-Aldrich, WHA1825047) using a vacuum filtration system, dried the filters at 105 °C for 2 h, and determined the TSS contents from the weight difference. From all wastewater samples, we prepared extracts containing dissolved extracellular enzymes (defined here as enzyme pool I) by centrifugation of 20 mL of raw wastewater (5 min, 2000*g*) and sterile syringe filtration (0.2 μm PES Sartorius, 16532K). Extracts containing both dissolved extracellular enzymes and EPS-bound extracellular enzymes (defined here as enzyme pool II) were prepared by adding 2 g of cation-exchange resin (CER) (Sigma-Aldrich, 91973) to 20 mL of raw wastewater and horizontally shaking these suspensions at 200 rpm for 30 min prior to centrifugation. Otherwise, the extraction process was the same for pools I and II. All extractions were performed in triplicate, and extracts were kept on ice until further use.

### Characterization of Wastewater Extracts

We determined protein concentrations using the Pierce bicinchoninic acid (BCA) protein assay kit (Thermo Fisher, 23225) according to the manufacturer’s instructions. In brief, we added 150 μL of enzyme extract or protein standard solution (bovine serum albumin, final protein concentration range 0–400 μg/mL) to 150 μL of the working reagent in a transparent 96-well plate. After 2 h of incubation at room temperature in the dark, we measured absorbance at 562 nm using a Tecan Infinite 200 pro plate reader. For peptidase activity measurements, we used the EnzChek protease assay kit (Thermo Fisher, E6638), which contains fluorogenic casein as substrate.^33,34^ The working solution was prepared according to the manufacturer’s instructions. We then mixed 100 μL of the enzyme extract and 100 μL of the freshly prepared working solution into a well of a black 96-well microplate (Eppendorf, Microplate 96/U-PP, black wells). Fluorescent hydrolysis products were quantified using a Tecan Infinite 200 pro plate reader (excitation: 485 nm; emission: 530 nm; interval of measurement: 4 min).

### AMP Incubation Experiments

We thawed enzyme extracts and transferred 1.35 mL to 2 mL protein LoBind Eppendorf tubes. For autoclaved controls, extract aliquots of 1.5 mL were autoclaved for 20 min at 121 °C and 2 bar (Wolf Sanoclav LaS-MCS-J) and 1.35 mL of the autoclaved extracts were transferred into 2 mL protein LoBind Eppendorf tubes. We then spiked 150 μL of the AMP stock solution containing all AMPs at 10 μg/mL to the enzyme extracts or 1.35 mL of ultrapure water. To demonstrate that none of the tested AMPs was present in non-spiked wastewater samples, we ran a control incubation in which we spiked 150 μL of ultrapure water to 1.35 mL of the enzyme extracts. Triplicate incubations were conducted for the autoclaved and active extracts with spiked AMP. After AMP spiking, we mixed the solutions by inverting the vials and incubating them at 20 °C under horizontal shaking at 300 rpm. To stop enzymatic reactions at pre-defined sampling time points (i.e., 2 min, 30 min, 1 h, 2 h, and 4 h), we transferred 150 μL of the incubation solution to 450 μL of acetonitrile containing 1% (v/v) formic acid in 1.5 mL protein LoBind Eppendorf tubes, mixed the resulting solution by inverting the tube and by horizontally shaking it at 300 rpm for 2 min at 20 °C, and centrifuged samples at 16,000*g* for 1 min. This protocol step was conducted according to Luther et al.^9^ We then transferred the supernatant into a fresh 1.5 mL protein LoBind Eppendorf tube and dried it in a SpeedVac vacuum concentrator (30 °C for approximately 6 h). To resolubilize AMPs, we added 150 μL of water containing 5% (v/v) acetonitrile and 0.1% (v/v) formic acid, vortexed the samples, and centrifuged them for 10 s at 1000*g* at room temperature. We transferred the solution into QuanRecovery HPLC vials (Waters, 186009186) and stored the samples at −20 °C until analysis. For method development, selected (and indicated) samples were sonicated (Sonorex Super RK 106, Bandelin, max 480 W) for 5 min at different steps of the sample preparation process.

### HPLC–HRMS Analysis

We analyzed AMPs using UHPLC (Thermo Scientific Dionex Ultimate 3000) equipped with a Waters XSelect PREMIER CSH C18 column (article number: 186009870) coupled to HRMS (Thermo QExactive). We injected 10 μL of each sample and applied a flow rate of 0.4 mL/min using the following eluent gradient (A: ultrapure water, B: LCMS-grade acetonitrile; both containing 0.1% LCMS-grade formic acid): 0–1 min: 2.5% B, 1–7 min: 2.5% B–40% B, 7–10 min: 40% B–95% B, 10–11 min: 95% B, 11–14 min: 95% B–2.5% B, 14–17 min: 2.5% B. Detection parameters were chosen as follows: MS full-scan: range: 100–1500 *m*/*z*, resolution: 140,000, AGC target: 1 × 10^6^, maximum IT: 100 ms, positive electrospray ionization (tune data: capillary temperature: 275 °C, sheath gas: 15, aux gas: 10, sweep gas: 1, S-lens RF: 50.0), MS/MS acquisitions: Top5, resolution: 17,500, AGC target: 1 × 10^5^, maximum IT: 50 ms, isolation window: 1.0 *m*/*z*, NCE (stepped): 10, 20, 30, dynamic exclusion time: 2.0 s. We used Skyline (version 20.2.0.343) to analyze the raw data. Criteria for parent peptides and hydrolysis product identification were set as described before:^31^*m*/*z* deviation <2 ppm, MS/MS fragments with *m*/*z* deviation <5 ppm, and reasonable chromatographic peak shape. For products, the peak area had to either increase throughout the incubation experiment or first increase and then decrease. We screened the HRMS data for sodium adducts of all AMPs but did not detect any.

From the progress curves of the incubation experiments, we estimated the biotransformation extent as described in eq 1 (where *C*_biot_: concentration in active extracts, *C*_abiot_: concentration in autoclaved extract)
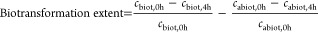
1

## Results and Discussion

### AMP Selection and Development of HPLC–HRMS Method

We selected a set of 10 AMPs based on recent key literature. Some AMPs were selected due to their described antimicrobial potential (e.g., cecropin P1, R8, tachyplesin I, murepavadin derivates, omiganan, and melittin),^8−10,38,39^ while others were included in the study because they have already been in commercial use as antibiotics (e.g., daptomycin, colistin A and B, and bacitracin A),^40−42^ or because initial insights into their environmental fate have been gained (e.g., daptomycin, colistin A and B, and bacitracin A).^14,33,43^ At the same time, we selected AMPs with the goal of covering a broad range of chemical diversity (i.e., linear vs cyclic peptides, different charge states, and canonical and non-canonical amino acids).

We developed an analytical method based on HPLC–HRMS to quantify all selected AMPs in a single measurement (analytical parameters including detected *m*/*z*, retention times, charge states at neutral pH, and detected MS2 fragments are provided in Tables S4 and S5). By injecting AMPs at varying concentrations (ranging from 1 μg/L to 1 mg/L) onto three different reversed-phase LC columns {i.e., two conventional C18 columns with different column lengths and particle sizes [i.e., Agilent InfinityLab Poroshell 120 Bonus-RP (2.7 μm, 2.1 × 150 mm) and Agilent Eclipse Plus C18 RRHD (1.8 μm, 2.1 × 50 mm)] and one C18 column that was specifically designed for basic compounds, i.e., Waters XSelect Premier CSH C18 (2.5 μm, 2.1 × 150 mm)}, we found that using the C18 column for basic compounds yielded overall the best chromatographic separation with good retention times, signal intensities, and peak shapes for all tested AMPs (Figure S3).

We further tested whether the material of the LC vial influences the signal intensities of our analytes (e.g., if analytes adsorb to the vial surface). Therefore, we determined the relative recovery of AMPs during our experimental procedure using three different LC vial types (i.e., uncoated glass inserts, an enhanced glass surface, and functionalized polypropylene). We found the highest recoveries when using functionalized polypropylene vials (Figure S4). Therefore, we consistently measured all samples in these vials.

### Extracellular Peptidase Activity across Treatment Stages and WWTPs

To assess extracellular peptidase activity profiles along communal wastewater treatment processes, we took samples at different treatment stages. We obtained samples from four tested full-scale WWTPs and prepared extracts of extracellular enzymes according to a previously developed method.^33,44^ This extraction method results in two pools of enzymes: enzymes that are dissolved in the extracellular solution (pool I) and total extracellular enzymes (i.e., dissolved extracellular enzymes as well as enzymes bound to the EPS; pool II).

**Figure 1:**
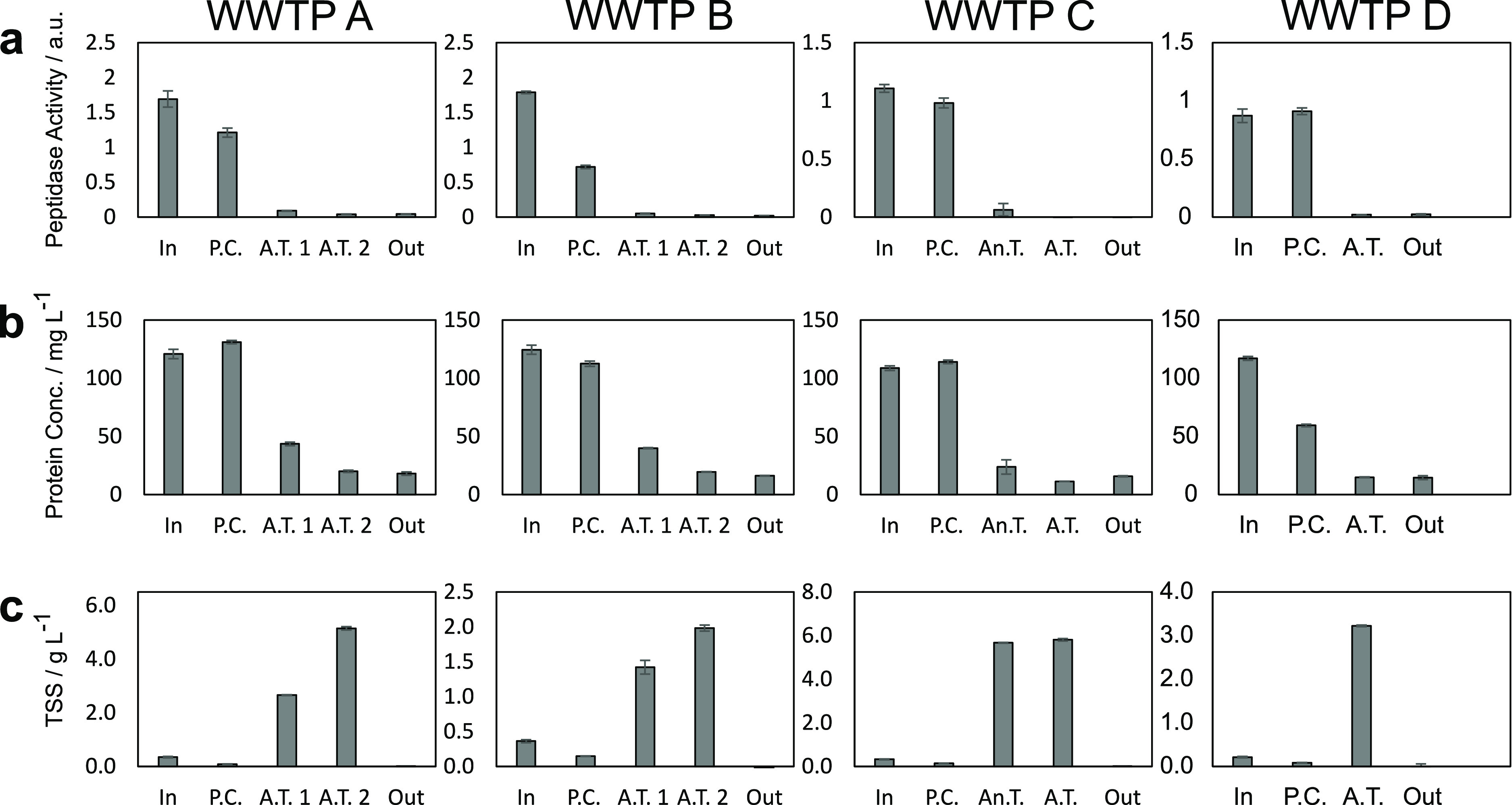
Peptidase activity (a) and protein concentration (b) of dissolved extracellular extracts (pool I) and TSS contents (c) of wastewater samples. Samples were obtained from different stages of four different full-scale WWTPs (WWTPs, In—influent, P.C.—primary clarification, A.T.—aeration tank, An.T.—anaerobic tank, and Out–effluent). Data points and error bars represent means ± standard deviations of triplicate extractions.

To gain insights into the peptidases in these extracts, we first measured general peptidase activity using a fluorescence-based assay.^33,37^ For both enzyme pools (i.e., I and II) and consistently across the four tested WWTPs, we found higher peptidase activities in extracts from raw wastewater (i.e., influent) and primary clarifiers than in extracts from bioreactors (Figures 1a, and S5). The decrease in activity between the primary clarifier and first bioreactor was more pronounced for enzyme pool I than for enzyme pool II, which can be explained by the adsorption of dissolved enzymes to the EPS or by the preferential inactivation (e.g., through hydrolysis) of dissolved enzymes compared to EPS-bound enzymes between these steps. We further assessed peptidase activities in non-processed wastewater grab samples from the influent and aeration tank of WWTP A. Peptidase activities were twice as high in influent samples compared to aeration tank samples. When comparing the peptidase activities of the non-processed grab samples to the respective pool I extracts, we found that in influent samples, only approximately one-third of the activity was pellet-associated, while in aeration tanks, almost all peptidase activity was pellet-associated (Figure S6). Based on the high peptidase activity in influent pool I samples (Figures 1a, and S5), and because an early extracellular inactivation of antimicrobials can be considered beneficial concerning resistance evolution, we chose to perform AMP biotransformation experiments with pool I extracts from influent samples.

Complementary to peptidase activity, we also quantified protein concentrations in all extracts and found a strongly decreasing protein concentration along the treatment process for all tested WWTPs (Figures 1b and S5). Lastly, we measured the TSS content (used as a rough proxy for biomass in wastewater bioreactors^45^) of all samples and found substantially lower TSS in raw wastewater and primary clarification tanks compared to bioreactors (Figure 1c). We further sampled WWTP A four times from May to December and determined the peptidase activity, protein concentration, and TSS for pools I and II as described above. We consistently found that extracellular peptidase activity and protein concentration are highest in influent samples, whereas TSS contents are highest during the biological treatment (Figure S7).

Our observation that the protein concentration in pool I was in some cases higher than the protein concentration in the corresponding pool II sample suggested that some proteins adsorbed to the CER that is used in the EPS disruption step of the extraction of enzyme pool II. We confirmed the adsorption of proteins to CER by showing a decrease in protein concentration upon incubating pool I extracts with CER (Figure S8). Due to the adsorption of proteins to CER, a comparison of peptidase activities and protein concentrations between pool I and pool II is not possible. However, it is noteworthy that an additional incubation of pool II extracts with CER did not result in a substantial decrease in the protein concentration (Figure S8), which is likely explained by variable adsorption tendencies across proteins. This has not been reported in previous studies in which the extraction method was exclusively applied to bioreactor systems, where protein concentrations in pool II extracts largely exceeded protein concentrations in pool I extracts despite the adsorption of proteins to CER reported here.^33,44^

### Kinetics of AMP Biotransformation

To assess the potential of wastewater peptidases to hydrolyze AMPs, we incubated the 10 selected AMPs with enzyme extracts (pool I) from WWTP influents because these extracts showed the highest extracellular peptidase activities, and we thus expected the highest potential for AMP hydrolysis in these extracts. We tested different protocol variations for sampling at distinct time points during incubations and to stop the enzymatic reaction while trying to maximize AMP recovery. In the standard protocol,^9,46^ proteins were precipitated in acetonitrile containing 1% formic acid. When the samples were solely acidified with formic acid (final concentration: 0.1%), the recovery for several AMPs (i.e., colistin A, daptomycin, omiganan, and R8) strongly decreased (Figure S9). When we entirely renounced precipitation and acidification, we observed better recovery for daptomycin and murepavadin; however, for cecropin P1, colistin A, omiganan, and R8, the recovery was much lower compared to the standard protocol (Figure S9)—presumably due to continued enzyme activity. To test whether sonication improves the recovery of AMPs, we added a sonication step at different stages of the standard protocol (i.e., before precipitation, during precipitation, and directly before analysis by LC–MS). We found that sonication helped to improve the recovery of some AMPs, but when considering all AMPs, the standard protocol without sonication yielded the best results (Figure S10).

In parallel to AMP incubations with active enzyme extracts, we conducted AMP incubations with autoclaved enzyme extracts to control for abiotic removal (e.g., due to adsorption to components in wastewater extracts). Autoclaving decreased the peptidase activity, as measured with the above-mentioned fluorogenic probe-based assay, of wastewater extracts to <3% of their original activity. To assess AMP stability in water without wastewater components, we spiked AMPs to ultrapure water (pH 6.8) and conducted incubation experiments akin to incubation experiments with wastewater extracts. We found that the AMP concentrations remained stable during their incubation with ultrapure water (Figure S11). We determined the AMP concentrations in unspiked enzyme extracts, which were below the limit of quantification (LOQ) for all AMPs in all WWTPs.

**Figure 2:**
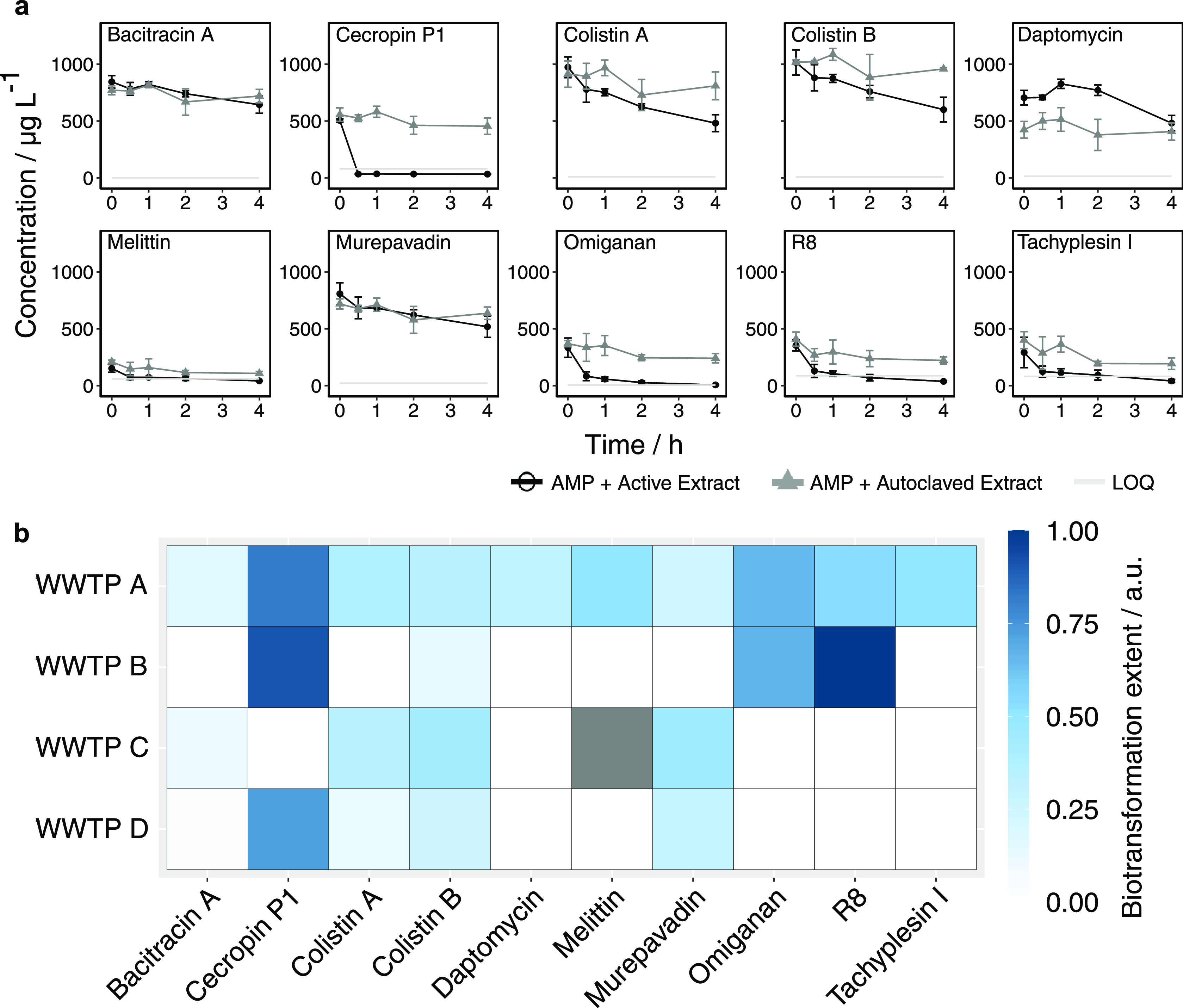
a) Progress curves of AMP concentrations during their incubation with active and autoclaved dissolved extracellular wastewater extract (i.e., pool I, see text) from WWTP A. Data points and error bars represent mean ± standard deviations of triplicate incubations. For colistin, the sum of colistin A and B was 1000 μg/L, with an unknown A/B ratio. Therefore, no *y*-axis label is provided for these two AMPs. (b) Biotransformation extents were calculated according to eq 1. The color in each cell represents the means of triplicate incubations. We additionally applied *t* tests to identify the level of statistical significance for differences in AMP concentration between the first and last time point in active extracts. AMP–WWTP combinations with *p* > 0.05 are shown in white. Gray cell represents value that could not be calculated because AMP concentration was below the LOQ.

Progress curves of AMP concentrations in active and autoclaved wastewater extracts are shown in Figure 2 (WWTP A) and Figure S12 (all four tested WWTPs). Melittin and tachyplesin I were rapidly removed upon addition to autoclaved enzyme extracts from all WWTPs, except for WWTP A in the case of tachyplesin I (Figures 2 and S12). Due to this finding, which we ascribe to rapid adsorption of these two AMPs to components in the wastewater extracts, we could not draw any conclusions about the biotransformation of melittin and tachyplesin I by extracellular wastewater enzymes. For the remaining eight AMPs, measured concentrations at the initial sampling time point were, in many cases, also substantially lower than the spiked concentrations—an observation that might again be explained by rapid adsorption to matrix components. However, the concentrations of these eight AMPs were still sufficiently high at initial sampling time points—and at similar initial levels between incubations in autoclaved and active extracts—so that an assessment of biotransformation was possible.

Regarding their susceptibility to biotransformation by enzymes in wastewater extracts, we found remarkable differences across the tested AMPs and remarkable similarities between the tested WWTPs. To visualize these results (Figure 2b), we calculated biotransformation extents based on concentration progress curves (Figure S12) using eq 1.

Cecropin P1, R8, and omiganan were rapidly removed to nonquantifiable concentrations by enzymes in active extracts from all tested WWTPs. Cecropin P1 was only detected at the first sampling time point (i.e., directly after spiking), while after 30 min of incubation, its concentration was below the LOQ in active extracts from all tested WWTPs—representing a detectable removal of up to 85% between these sampling points. R8 and omiganan also reached nonquantifiable concentrations during the incubations—representing a detectable removal of up to 85 and 98%, respectively, of the amounts detected at the first time point. Cecropin P1, R8, and omiganan are linear peptides that entirely consist of canonical amino acids, which are both factors that might explain the observed rapid biotransformation by enzymes in wastewater extracts.

Colistin A and B were also biotransformed by enzymes in wastewater extracts—as shown by a more extensive decrease in concentration during their incubation with active extracts than with autoclaved extracts. However, biotransformation of both colistins was slower than that of cecropin P1, R8, and omiganan; the detectable removal during incubation was up to 50 and 60%, respectively, of the initially detected amounts.

For murepavadin, bacitracin A, and daptomycin, we did not observe substantial removal due to biotransformation as concentrations in active and autoclaved extracts behaved very similarly during incubation for these three compounds (Figures 2 and S12). Our results on daptomycin are consistent with an earlier study that reported daptomycin concentrations to be stable during its incubation with dissolved extracellular wastewater enzymes derived from aeration tanks of WWTPs.^33^ We note that daptomycin concentrations in experiments with autoclaved controls were lower compared to active extracts for WWTPs A, B, and C, which might be explained by increased adsorption of daptomycin to components in autoclaved—compared to active—wastewater extracts (Figure S12). Murepavadin, bacitracin A, and daptomycin are cyclic and contain non-canonical amino acids—both factors might explain why these compounds were not biotransformed in wastewater extracts.

### Pathways of Enzymatic AMP Hydrolysis

To learn about the enzymatic hydrolysis pathways of AMPs that we found to be rapidly biotransformed by enzymes in wastewater extracts, we predicted all products of single peptide bond hydrolysis reactions and screened our data-dependent high-resolution mass spectrometry data for experimental evidence in support of the predicted hydrolysis products. These analyses were conducted for the AMPs with the highest biotransformation extents (i.e., >0.5 in at least two WWTPs), namely, cecropin P1, omiganan, and R8. The following requirements for transformation products (TPs) were defined: at least three amino acids, peak area either increases during the incubation experiment or first increases and then decreases, peak area exceeds that in AMP-free enzyme extract by >5-fold and that in spiked autoclaved extracts by >6-fold, and mass deviation <2 ppm from the calculated exact mass. Mass lists, mass deviations, and retention times of all TPs can be found in Tables S6–S11. We further predicted TPs formed via exocleavage mechanisms (i.e., cleavage of one or two terminal amino acids simultaneously from the C- and N- terminus of the peptide) and screened for these TPs in the HRMS data. However, we did not find evidence of the formation of these products.

**Figure 3:**
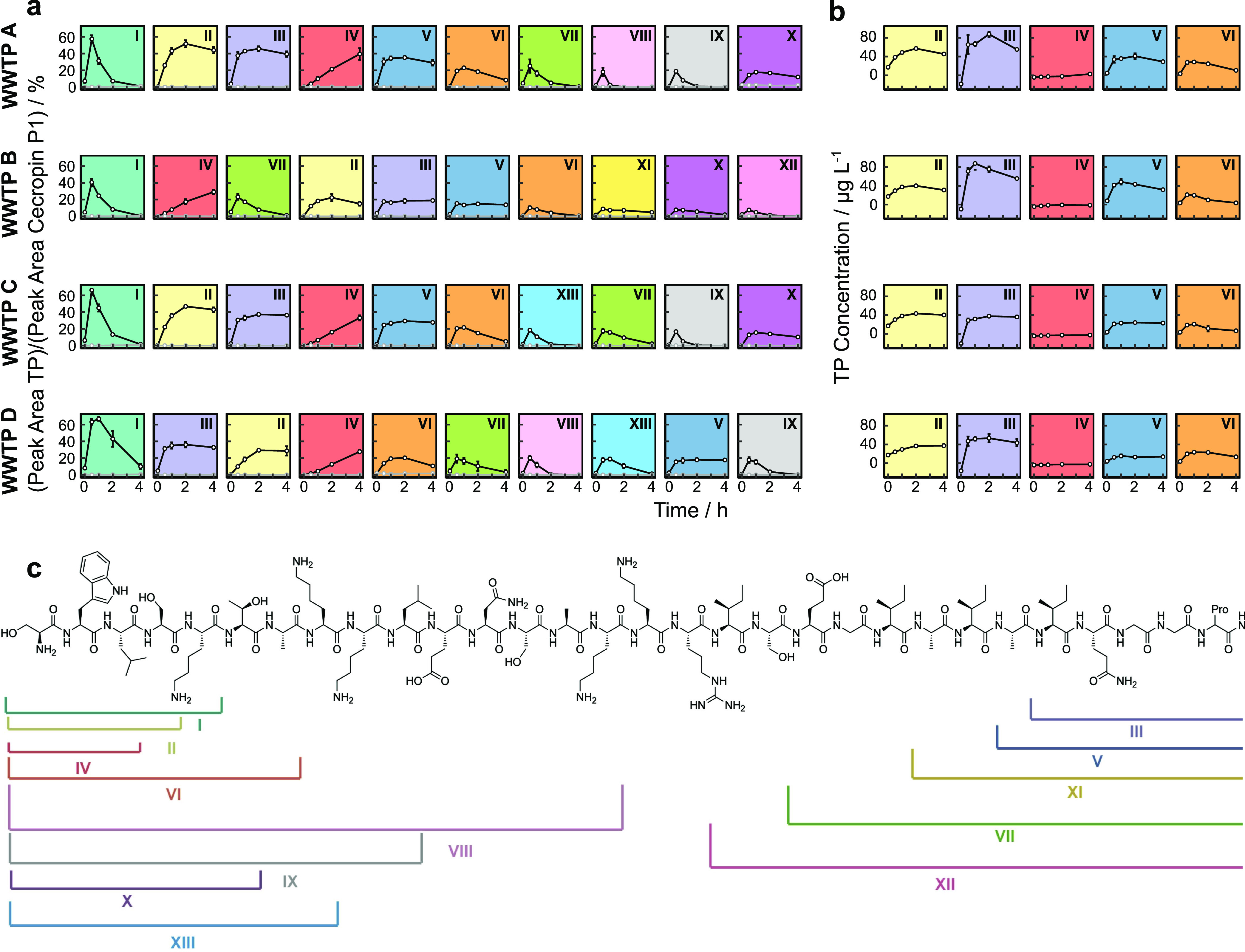
(a) Progress curves of the most intensely detected transformation products (TPs) of cecropin P1 during its incubation in dissolved extracellular wastewater extracts. Peak areas of TPs at each time point were divided by the peak area of cecropin P1 calibration at a concentration of 1 mg/L. Data points and error bars represent means ± standard deviations of triplicate incubations. (b) Absolute quantification of selected TPs was done using synthetic peptide standards. Data points and error bars represent means ± standard deviations of triplicate incubations. (c) Chemical structure of cecropin P1. Brackets show the identified TPs, indicated with roman numerals.

For each of these three AMPs and each WWTP, we ranked the identified products regarding the maximum peak area that was detected during the incubation of the AMPs with the active wastewater extract. For cecropin P1, 58 TPs were predicted in total. Of these products, we found evidence for 25 TPs in our experiments. We selected the 10 TPs with the highest peak areas per WWTP, which yielded a total of 13 individual TPs ranging in size from 3 to 15 amino acids (Figure 3). TP I had the highest peak area in all WWTP extracts, and almost all of the 13 TPs occurred in all WWTP extracts. Remarkably, not only the TPs but also the progress curves of their peak area were conserved across the four WWTP extracts, indicating that peptidases of similar specificities occur in all tested wastewaters. Similarly to cecropin P1, TP prediction for R8 yielded 38 individual TPs and we found evidence for 20 of these TPs in our experiments. Selecting the 10 most intense TPs per WWTP, we found 13 individual TPs containing 3 to 18 amino acids. 8 out of the 13 TPs are formed in extracts from all WWTPs, and the formation kinetics in the extracts are similar for the distinct TPs (Figure S13). For omiganan, we predicted a total of 18 TPs and found 9 of these TPs in our experiments, again with high similarity across the four WWTPs, but the TPs had much lower peak areas than TPs of R8 and cecropin P1 (Figure S14).

For the most stable TPs (i.e., TPs with high peak areas at the end of the incubation experiments), we performed absolute quantification using synthetic peptide standards. Exact masses, retention times, MS2 fragments, and mass deviations of these TPs are shown in Table S1. For cecropin P1, we performed absolute quantification for TPs II, III, IV, V, VI, and X and found the highest concentrations for TP III. Up to 8.8% of spiked cecropin P1 is converted to TP III during incubations with wastewater extract. For TP II, the conversion ranges from 3.7–5.7%, and for TP V and VI, conversions range between 1.5 and 4.9 and 2.1 and 2.9%, respectively. For TPs IV and X, less than 1% of cecropin P1 was converted to these products. We ascribe the differences in the rank order between relative signal intensity and absolute concentrations to different ionization efficiencies, which highlights the importance of absolute product quantification for biotransformation studies. For R8, we performed absolute quantification for TPs I, II, IV, V, and VII. Up to 7.8% of R8 is biotransformed to product I. For all other products, we found conversions of less than 3.2%. Similar to cecropin P1, the rank orders based on absolute concentrations and peak areas were different also for R8. For omiganan, we only conducted absolute quantification for TP I, which resulted in less than 1% of omiganan being converted to TP I. This suggests that other, non-detected, TPs were the predominant biotransformation routes.

In total, the six quantified TPs for cecropin P1 sum to 22.8% of the initially spiked AMP concentration. Considering that up to 70% of the spiked cecropin P1 is removed by sorption and might thus be less available for biotransformation, the found TPs account for a substantial part of the nonsorbed cecropin P1. To investigate the adsorption of the TPs to the wastewater matrix, we conducted incubation experiments using active and autoclaved pool I extracts from the influents of WWTP A and D with varying levels of AMP sorption (Figure S15). We quantified the sorption potential in the WWTP extracts by comparing the TP concentration in spiked autoclaved wastewater extracts to that in spiked ultrapure water (Table S12). For the TPs of cecropin P1, we found that up to 61% of the spiked TP concentration is not detectable in wastewater extracts—most likely due to sorption. We further found that the individual TPs show large variations in their sorption potential (Table S12). Without considering sorption, we detected 22.8% of the spiked cecropin P1 concentration transformed by wastewater hydrolases to six selected TPs. When considering the maximum sorption potential for each TP, we derived from the experimental data that up to 31.6% of the spiked cecropin P1 concentration could be transformed into the six selected TPs. To complete the mass balance, simultaneous hydrolytic events that result in different TPs and other biotransformation pathways (i.e., oxidation and hydroxylation) would need to be considered. For R8, we found similar trends: the five quantified TPs sum to 17.7% of the spiked R8 concentration. By spiking these five TPs into wastewater extracts, we found that up to 46.7% of the TPs are removed—most likely due to sorption processes. Considering the maximum sorption potential of each TP, we derived that the five TPs could account for 22.8% of the initially spiked R8 concentration (Table S12).

### Environmental Implications

Our finding that the highest extracellular dissolved peptidase activities occurred early in the wastewater treatment process indicates that some peptide-based chemicals might be hydrolyzed in the sewer system and thus independently of wastewater treatment infrastructure. This finding also raises questions regarding the origin of the peptidases in wastewater. Such peptidases might be secreted by microbes present in wastewater, originate from human or animal wastes, or be part of home care products, such as laundry detergent formulations.

Concerning the hydrolyzability of AMPs by peptidases in raw wastewater, we showed that 3 out of 10 tested AMPs were readily biotransformed in extracellular dissolved extracts from at least 2 WWTP influent samples. By analyzing the TPs of these three most susceptible AMPs, we found a high similarity of TPs after the incubation with extracts from different WWTPs as well as similar formation and stability trends, suggesting that certain peptidase specificities are conserved across sewer systems. Whether this biotransformation also inactivates AMPs is yet to be understood, but we found that the products of the highest signal intensities are much smaller than the parent AMP. Besides biotransformation, our results indicated rapid adsorption of several AMPs to components in wastewater extracts. The effect of adsorption on the fate of AMPs during the wastewater treatment process and in downstream environments remains to be investigated.

## Supporting Information Available

Chemical structures of AMPs; AMP calibration curves; AMP chromatographs on tested columns; AMP recoveries using different vials; peptidase activities, protein concentrations, and total suspended solid contents across WWTPs and across seasons; peptidase activities in raw wastewater and extracts; sorption to CER; optimization of sample preparation; effect of sonication on recovery; stability of AMPs in ultrapure water; AMP concentration curves in wastewater extracts; TPs of R8 omiganan; experiments with TPs; TPs; WWTP parameters; pH values of wastewater extracts; analytical parameters of AMPs; MS2 fragments of AMPs; overview of TPs for cecropin P1, R8, and omiganan; and sorption of TPs (PDF)
