# Supplementary material for: Hydrolysis of Antimicrobial Peptides by Extracellular Peptidases in Wastewater
Source: Environ Sci Technol. 2023 Dec 16;58(1):717–26. doi: 10.1021/acs.est.3c06506 (PMC10785756; doi:10.1021/acs.est.3c06506)
Supplement: Supplementary file 1 — es3c06506_si_001.pdf [file es3c06506_si_001.pdf]

Supporting Information

Hydrolysis of antimicrobial peptides by extracellular peptidases in wastewater

Natalie Wichmann[1,2], Richard Gruseck[1], and Michael Zumstein[1*]

[1]Division of Environmental Geosciences, Centre for Microbiology and Environmental Systems Science, University of Vienna, Josef-Holaubek-Platz 2, 1090 Vienna, Austria

[2] Department of Environmental Microbiology, Swiss Federal Institute of Aquatic Science and Technology (Eawag), Überlandstrasse 133, 8600 Dübendorf, Switzerland

[*]To whom correspondence should be addressed:

E-mail: michael.zumstein@univie.ac.at

This PDF file includes:

Figure S1. Chemical structures of selected antimicrobial peptides (AMPs).

Figure S2. Calibration curves for the selected antimicrobial peptides (AMPs).

Figure S3. Chromatographs of antimicrobial peptides (AMPs) recorded on three different reverse phase C18 columns.

Figure S4. Antimicrobial peptide (AMP) recovery using different liquid chromatography vials.

Figure S5. Peptidase activity (a) and protein concentration (b) of protein extracts and total suspended solid contents (TSS, c) of raw wastewater samples.

Figure S6. Peptidase activities of wastewater grab samples and extracellular dissolved protein extracts (Pool I) from influent and aeration tank (A.T.) samples taken at a full-scale WWTP.

Figure S7. Seasonality of peptidase activity (a) and protein concentration (b) of protein extracts and total suspended solid contents (TSS, c) of raw wastewater samples.

Figure S8. Sorption of protein to the cation exchange resin (CER) used for the disruption of extra polymeric substance (EPS).

Figure S9. Control experiments stopping enzymatic processes to optimize the antimicrobial peptide (AMP) recovery.

Figure S10. Effect of sonication during sample preparation on antimicrobial peptide (AMP) recovery.

Figure S11. Stability of antimicrobial peptides (AMPs) in ultrapure water.

Figure S12. Progress curves of antimicrobial peptide (AMP) incubation with wastewater extracts from four full-scale wastewater treatment plants (WWTPs).

Figure S13. Transformation products (TPs) of antimicrobial peptide R8.

Figure S14. Transformation products (TPs) of antimicrobial peptide Omiganan.

Figure S15. Incubation of custom-synthesized transformation products (TPs) with ultrapure water, as well as active and autoclaved wastewater extracts from wastewater treatment plants A and D.

Table S1. List of custom-synthesized transformation products (TPs).

Table S2. Wastewater treatment plant (WWTP) parameters on respective sampling days.

Table S3. pH values of dissolved extracellular protein extracts (pool I) and extrapolymeric substance (EPS) -bound and dissolved extracellular protein extracts (pool II) at different stages of the wastewater treatment process.

Table S4. Analytical parameters of tested antimicrobial peptides (AMPs).

Table S5. MS2 fragments for tested antimicrobial peptides (AMPs).

Table S6. Overview of transformation products (TPs) for Cecropin P1.

Table S7. Validation of transformation products (TPs) of Cecropin P1 by MS2 data.

Table S8. Overview of transformation products (TPs) for R8.

Table S9. Validation of transformation products (TPs) of R8 by MS2 data.

Table S10. Overview of transformation products (TPs) for Omiganan.

Table S11. Validation of transformation products (TPs) of Omiganan by MS2 data.

Table S12. Sorption potential of the custom synthesized transformation products (TPs) to the wastewater matrix in wastewater treatment plants (WWTPs) A and D.

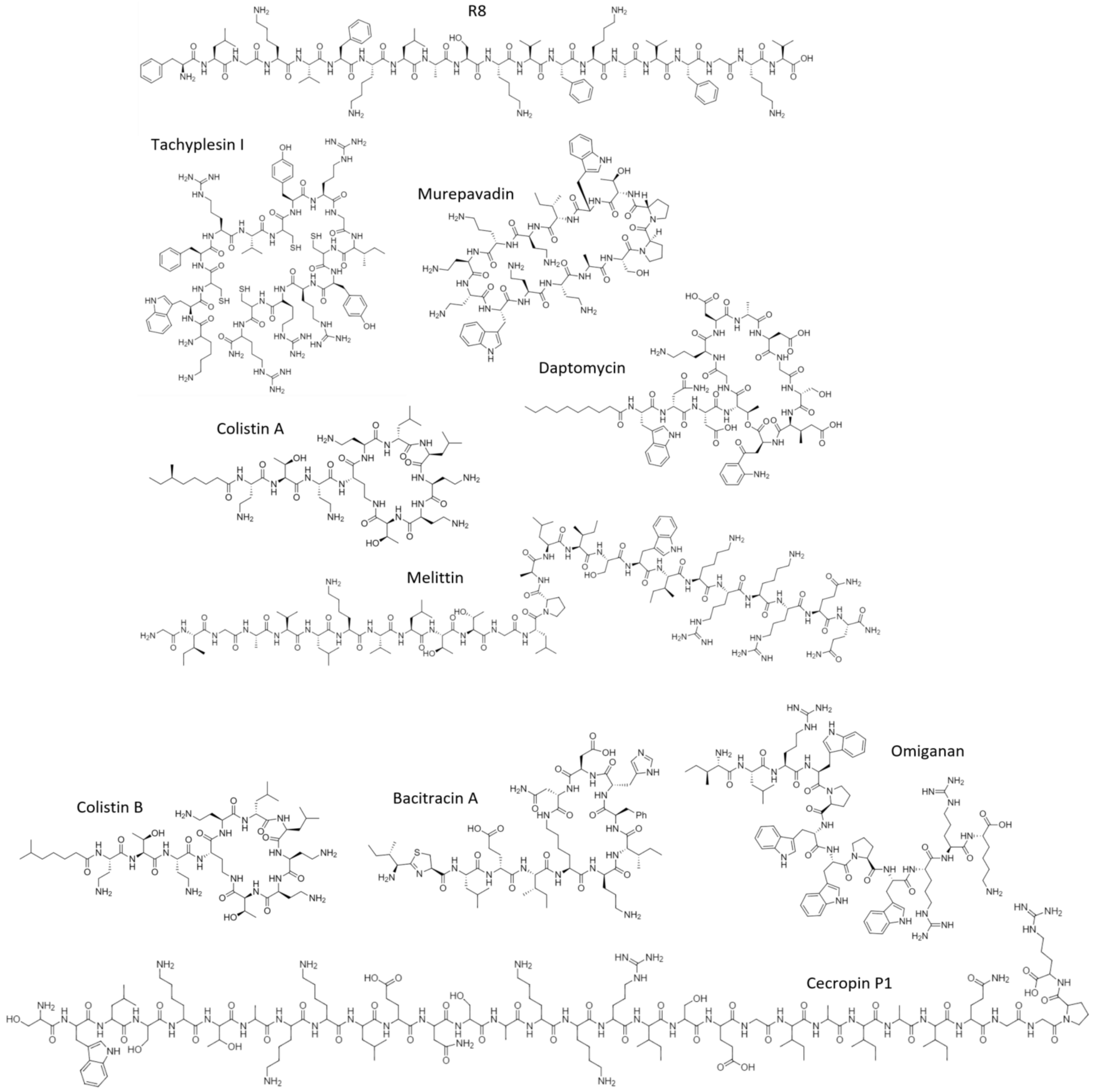

Figure S1. Chemical structures of selected antimicrobial peptides (AMPs).

Table S1. List of custom-synthesized transformation products (TPs): their exact and measured mass to charge ratios (m/z), the deviation of the exact and measured mass to charge ratios (m/z) and their retention times (RT) in minutes. MS2 fragments are noted in Roepstorff-Fohlmann-Biemann format[1,2] and exact m/z and measured m/z ratios, as well as their deviation (in ppm), are listed.

Transformation product	Exact m/z	Measured m/z	RT	Fragment	Exact m/z	Measured m/z	Deviation / ppm
$C_{28}H_{46}N_6O_6$	282.1812	282.1808	6.13	K y2	416.2867	416.2856	-2.64
				F a1	120.0808	120.0807	-0.83
				L a2	233.1648	233.1642	-2.57
$C_{37}H_{55}N_7O_7$	355.7154	355.7147	7.44	L y5	563.3552	563.3533	-3.37
				G y4	450.2711	450.2695	-3.55
$C_{17}H_{25}N_3O_4$	336.1918	336.1910	6.3	L y2	189.1234	189.1229	-2.64
				L b2	261.1598	261.1590	-3.06
$C_{30}H_{49}N_7O_7$	310.6919	310.6911	6.18	F y4	450.2711	450.2701	-2.22
				K y2	246.1812	246.1806	-2.44
$C_{27}H_{44}N_6O_6$	275.1734	275.1729	5.71	G y3	303.2027	303.2017	-3.30
				F y4	450.2711	450.2700	-2.44
$C_{23}H_{33}N_5O_7$	492.2453	492.2445	7.35	W b2	274.1186	274.1177	-3.28
				L y2	219.1339	219.1334	-2.28
$C_{26}H_{46}N_{10}O_8$	314.1823	314.1815	1.47	Q y5	514.2732	514.2718	-2.72
				G y4	386.2146	386.2132	-3.62
$C_{20}H_{28}N_4O_5$	405.2132	405.2125	7.70	W b2	274.1186	274.1176	-3.65
				W z2	301.1547	301.1533	-4.65
$C_{29}H_{51}N_{11}O_9$	349.7008	349.7004	1.93	Q y5	514.2732	514.2722	1.94
				G y4	386.2146	386.2135	2.85
$C_{36}H_{57}N_9O_{11}$	396.7162	396.7155	6.33	L y5	519.3137	519.3127	-1.93
				W b2	274.1186	274.1178	-2.92

$C_{33}H_{52}N_8O_{10}$	361.1976	361.1969	6.25	L y4	448.2766	448.2754	-2.68
				S y3	335.1925	335.1920	-1.49
$C_{61}H_{80}N_{14}O_9$	577.3189	577.3183	9.57	W b7	1038.5672	1038.5646	-2.50
				W b6	852.4879	852.4841	-4.46

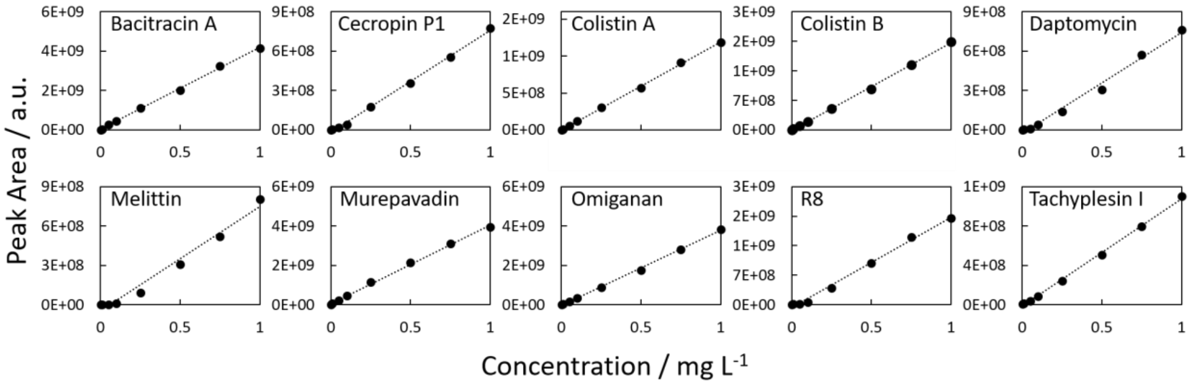

Figure S2. Calibration curves for the selected antimicrobial peptides (AMPs). Dilutions range from 0.001 mg/L to 1 mg/L. All AMPs were pooled for calibration. For Colistin, the sum of Colistin A and B was 1000 µg / L, with an unknown A/B ratio. Therefore, no x-axis label is provided for these two AMPs. Data points represent single measurements. The calibration was fitted using linear regression (dashed line).

Table S2. Wastewater treatment plant (WWTP) parameters on respective sampling days. EW60 refers to 60 g of biochemical oxygen demand that arise from the organic wastes produced per person and day. A.T.1 is the high-load aeration tank, while A.T.2 is the low-load aeration tank. For WWTP C, A.T.1 was anaerobic and the influent measurement system was defect; volumes are therefore based on average loads (indicated with asterisks*). Values were derived from average influent loads for dry weather. For WWTP D, there was only one aeration tank (A.T. 1). Hyphens indicate non-available data.

**WWTPs**				
**Parameter**	**WWTP A**	**WWTP B**	**WWTP C**	**WWTP D**
Sampling date	01.12.2021	20.07.2021	18.08.2021	01.09.2021
Influent volume on sampling day	6602.4 m^3	541840 m^3	5400 m^3 (*)	3679 m^3
Maximal capacity (EW60)	55000	4000000	30000	45000
Solid retention time A.T.1	0.6 d	0.6 d	-	45 d
Solid retention time A.T.2	6.2 d	9.4 d	-	-
Hydraulic retention time A.T.1	2.2 h	2.2 h	2.9 h (*, anaerobic tank)	13.4 h
Hydraulic retention time A.T.2	7.2 h	7.6 h	3.3 h (*, aerobic tank)	-
Influent Temperature	13.7 °C	20.1 °C	-	19.9 °C (effluent)
Influent pH	7.9	7.5	-	7.5
Type	Activated sludge	Activated sludge	Activated sludge	Activated sludge

Table S3. pH values of dissolved extracellular protein extracts (pool I) and extrapolymeric substance (EPS) -bound and dissolved extracellular protein extracts (pool II) at different stages of the wastewater treatment process (In – influent, P.C. – primary clarification, A.T. – Aeration tank, An.T. – anaerobic tank, Out – Effluent). Hyphens indicate non-available data caused by different operational modes of the WWTPs.

		**In**	**P.C.**	**A.T.1**	**An.T.**	**A.T.2**	**Out**
**WWTP A**	**Pool I**	8.6	8.5	8.1	-	7.8	7.9
	**Pool II**	9.0	8.9	8.4	-	8.3	8.3
**WWTP B**	**Pool I**	8.3	8.3	7.9	-	7.6	8.0
	**Pool II**	8.7	8.7	8.3	-	7.8	8.2
**WWTP C**	**Pool I**	8.3	8.3	-	8.0	7.9	8.2
	**Pool II**	8.9	8.8	-	8.5	8.6	8.8
**WWTP D**	**Pool I**	8.4	8.4	8.0	-	-	8.3
	**Pool II**	8.8	8.7	8.2	-	-	8.7

Table S4. Analytical parameters of tested antimicrobial peptides (AMPs): chemical formula, ionization state of detected precursor ion, exact mass to charge ratio (m/z), deviation (Dev.) of exact and measured mass to charge ratio in ppm, retention times (RT) in minutes and charge states at neutral pH conditions as well as at pH 8.4, which is the average pH of pool I enzymes in wastewater extracts (Table S3). For charge states, the Prot pi tool (https://www.protpi.ch/) was used. For cyclic AMPs and non-canonical amino acids, charge states were manually adapted.

AMP	Chemical Formula	Detected precursor ion	Exact m/z	Measured m/z	Dev. / ppm	RT/ min	Charge	
							pH 7	pH 8.4
Bacitracin A	$C_{66}H_{103}N_{17}O_{16}S$	$[M+3H]^{3+}$	474.9231	474.9233	0.42	8.2	-1.1	-1.9
Cecropin P1	$C_{147}H_{253}N_{45}O_{43}$	$[M+5H]^{5+}$	668.3872	668.3873	0.15	7.6	+4.4	+3.8
Colistin A	$C_{53}H_{100}N_{16}O_{13}$	$[M+3H]^{3+}$	390.5958	390.5958	0.00	6.1	+5.3	+4.8
Colistin B	$C_{52}H_{98}N_{16}O_{13}$	$[M+3H]^{3+}$	385.9239	385.9240	0.26	5.7	+5.3	+4.8
Daptomycin	$C_{72}H_{101}N_{17}O_{26}$	$[M+2H]^{2+}$	810.8625	810.8621	-0.49	11.1	-2.4	-3.0
Melittin	$C_{131}H_{229}N_{39}O_{31}$	$[M+5H]^{5+}$	569.9581	569.9585	0.70	9.2	+4.8	+4
Murepavadin	$C_{73}H_{112}N_{22}O_{16}$	$[M+4H]^{4+}$	389.2229	389.2231	0.51	4.8	+5.8	+4.9
Omiganan	$C_{90}H_{127}N_{27}O_{12}$	$[M+4H]^{4+}$	445.5112	445.5116	0.90	7.4	+3.8	+3.1
R8	$C_{111}H_{177}N_{25}O_{22}$	$[M+4H]^{4+}$	554.0948	554.0952	0.72	8.9	+4.5	+3.8
Tachyplesin I	$C_{99}H_{151}N_{35}O_{19}S_4$	$[M+5H]^{5+}$	453.4234	453.4235	0.22	6.1	+5.1	+2.6

Table S5. MS2 fragments for tested antimicrobial peptides (AMPs): exact mass to charge ratio (m/z), measured m/z, and deviation of m/z in ppm. Fragment ions of linear peptides containing exclusively canonical amino acids are noted according to Roepstorff-Fohlmann-Biemann nomenclature[1,2]. Fragment ions of cyclic peptides or peptides containing (some) non-canonical amino acids are noted as sum formula.

AMP	Fragment	Exact m/z	Measured m/z	Dev. / ppm
Bacitracin A	$[C_{32}H_{44}N_8O_8+H]^+$	669.3362	669.3365	0.45
	$[C_{40}H_{60}N_{12}O_{10}+H]^+$	869.4604	869.4625	2.42
	$[C_9H_{14}N_2OS+H]^+$	199.0897	199.0897	0.00
	$[C_{10}H_{14}N_2O_2S+H]^+$	227.0845	227.0845	0.00
Cecropin P1	Q y5	514.2732	514.2726	-1.17
	G y4	386.2146	386.2139	-1.81
	W b2	274.1186	274.1180	-2.19
	A y9	441.7614	441.7610	-0.91
	I y6	627.3573	627.3569	-0.64

	A y7	698.3944	698.3929	-2.15
	I b22	819.4710	819.4702	-0.98
	I b18	1035.6127	1035.6111	-1.54
	I b22	1228.7028	1228.7047	1.55
Colistin A	$[C_{13}H_{24}N_2O_2+H]^+$	241.1905	241.1906	0.41
	$[C_4H_8N_2O+H]^+$	101.07097	101.0712	2.28
Colistin B	$[C_{12}H_{22}N_2O_2+H]^+$	227.1755	227.1750	-2.20
	$[C_{20}H_{37}N_7O_5+H]^+$	456.2930	456.2922	-1.82
	$[C_4H_8N_2O+H]^+$	101.0712	101.0712	0.00
Daptomycin	$[C_{51}H_{73}N_{15}O_{24}+H]^+$	1280.5020	1280.5021	0.08
	$[C_{10}H_{10}N_2+H]^+$	159.0915	159.0915	0.00
	$[C_{20}H_{28}N_2O+H]^+$	313.2269	313.2271	0.64
	$[C_{21}H_{28}N_2O_2+H]^+$	341.2217	341.2220	0.88
Melittin	V b5	398.2398	398.2408	2.51
	T b11	1053.6667	1053.6663	-0.38
	V y19	736.7828	736.7838	1.36
	V b8	738.4872	738.4864	-1.08
Murepavadin	$[C_{19}H_{26}N_6O_3+H]^+$	387.2140	387.2144	1.01
	$[C_{15}H_{18}N_4O_2+H]^+$	287.1503	287.1501	-0.77
	$[C_9H_{18}N_4O_2+H]^+$	215.1503	215.1503	0.00
Omiganan	R b3	383.2765	383.2772	1.83
	L b2	227.1754	227.1756	0.88
	P b5	333.7079	333.7078	-0.30
	W b4	569.3558	569.3552	-1.05
R8	K y2	246.1812	246.1807	-2.03
	L b2	261.1598	261.1595	-1.15
	G y3	303.2027	303.2021	-1.98
	K b14	532.0008	532.0005	-0.56
	V y5	549.3395	549.3394	-0.18
	V b16	588.7027	588.7010	-2.89
	K b11	610.3817	610.3820	0.49
	A y12	640.8899	640.8899	0.00
	L y13	697.4319	697.4335	2.29
	F b6	692.4114	692.4130	2.31
	K y7	748.4716	748.4717	0.13
	K b7	820.5080	820.5087	0.85
	F y8	895.5400	895.5404	0.45
Tachyplesin I				

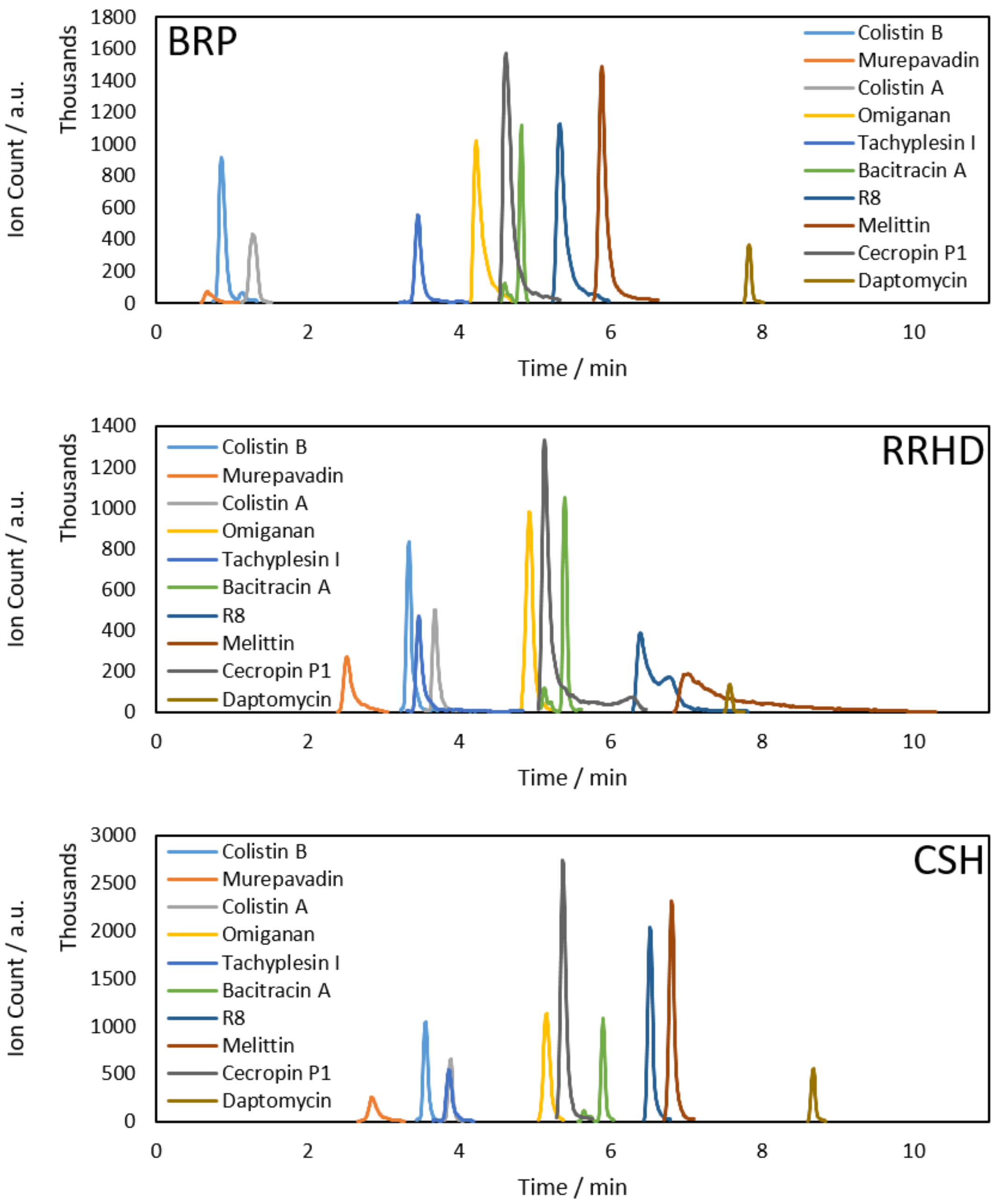

Figure S3. Chromatographs of antimicrobial peptides (AMPs) recorded on three different reverse phase C18 columns. Precursor ion counts were cut off at 1 % of their maximal peak height. BRP: Agilent InfinityLab Poroshell 120 Bonus-RP (2.7 µm, 2.1x150mm, article number: 693768-901). RRHD: Agilent Eclipse Plus C18 RRHD (1.8 µm, 2.1x50mm, article number: 959757-902). CSH: Waters XSelect Premier CSH C18 (2.5 µm, 2.1x150 mm) with Guard Column Van Guard XSelect Premier CSH C18 (article number: 186009870).

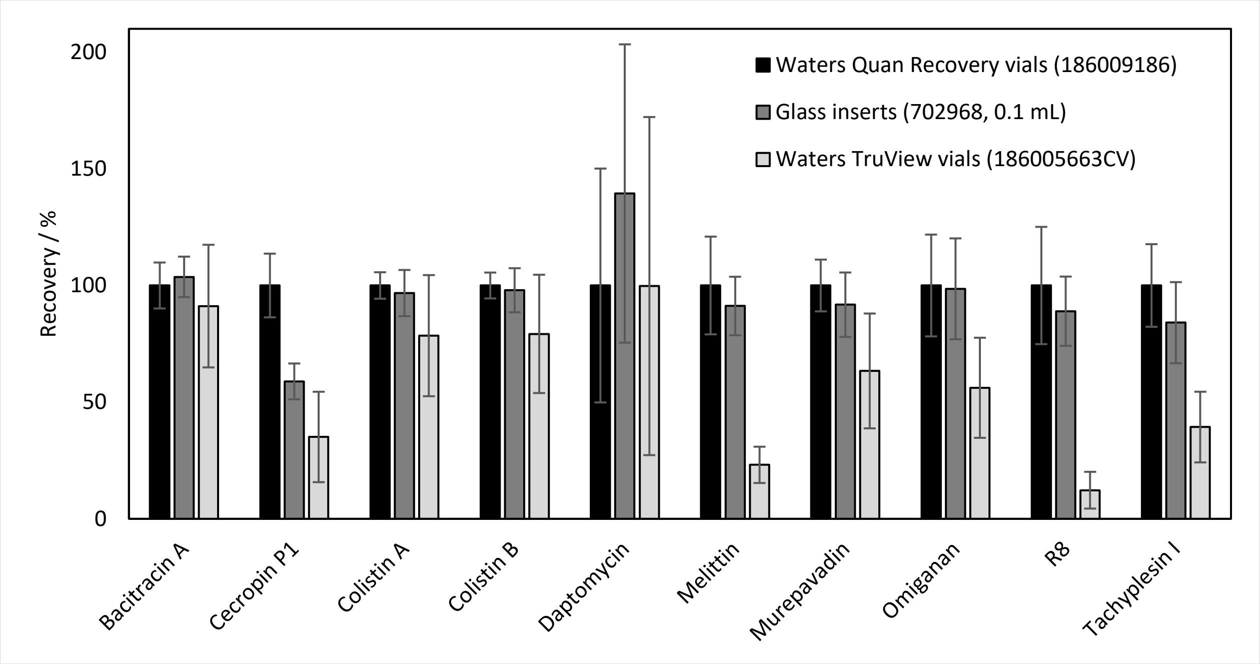

Figure S4. Antimicrobial peptide (AMP) recovery using different liquid chromatography vials. AMPs were spiked to ultrapure water and the same protocol as for the wastewater extract incubations was performed. Data points and error bars represent mean +/- standard deviations of triplicate incubations. All values are normalized to the peak area in Waters Quan Recovery vials for each AMP.

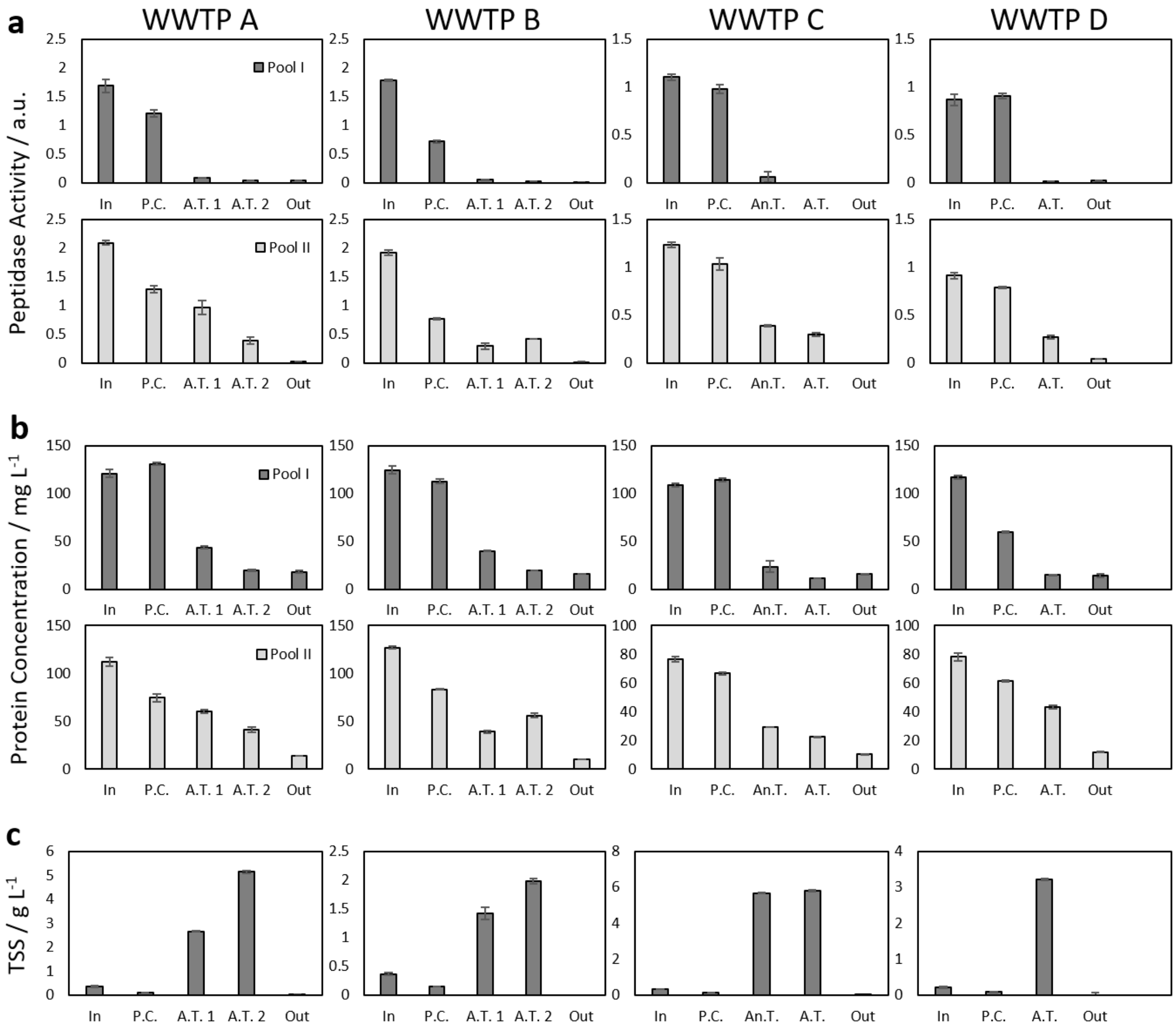

Figure S5. Peptidase activity (a) and protein concentration (b) of protein extracts and total suspended solid contents (TSS, c) of untreated wastewater samples. Pool I: Dissolved extracellular protein; Pool II: Extrapolymeric substance (EPS) -bound and dissolved extracellular protein. Samples were obtained from different stages of four different full-scale wastewater treatment plants (WWTPs, In – influent, P.C. – primary clarification, A.T. - Aeration tank, An.T. – anaerobic tank, Out – Effluent). Data points and error bars represent means +/- standard deviations of triplicate extractions.

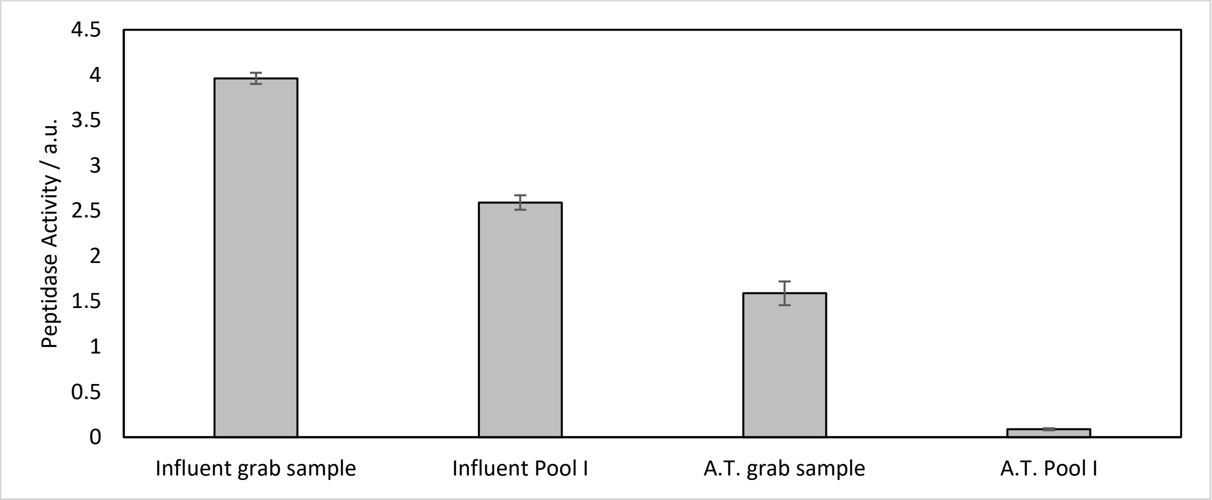

Figure S6. Peptidase activities of wastewater grab samples and extracellular dissolved protein extracts (Pool I) from influent and aeration tank (A.T.) samples taken at WWTP A. Data points and error bars represent means +/- standard deviations of triplicate sample aliquots and extractions, respectively.

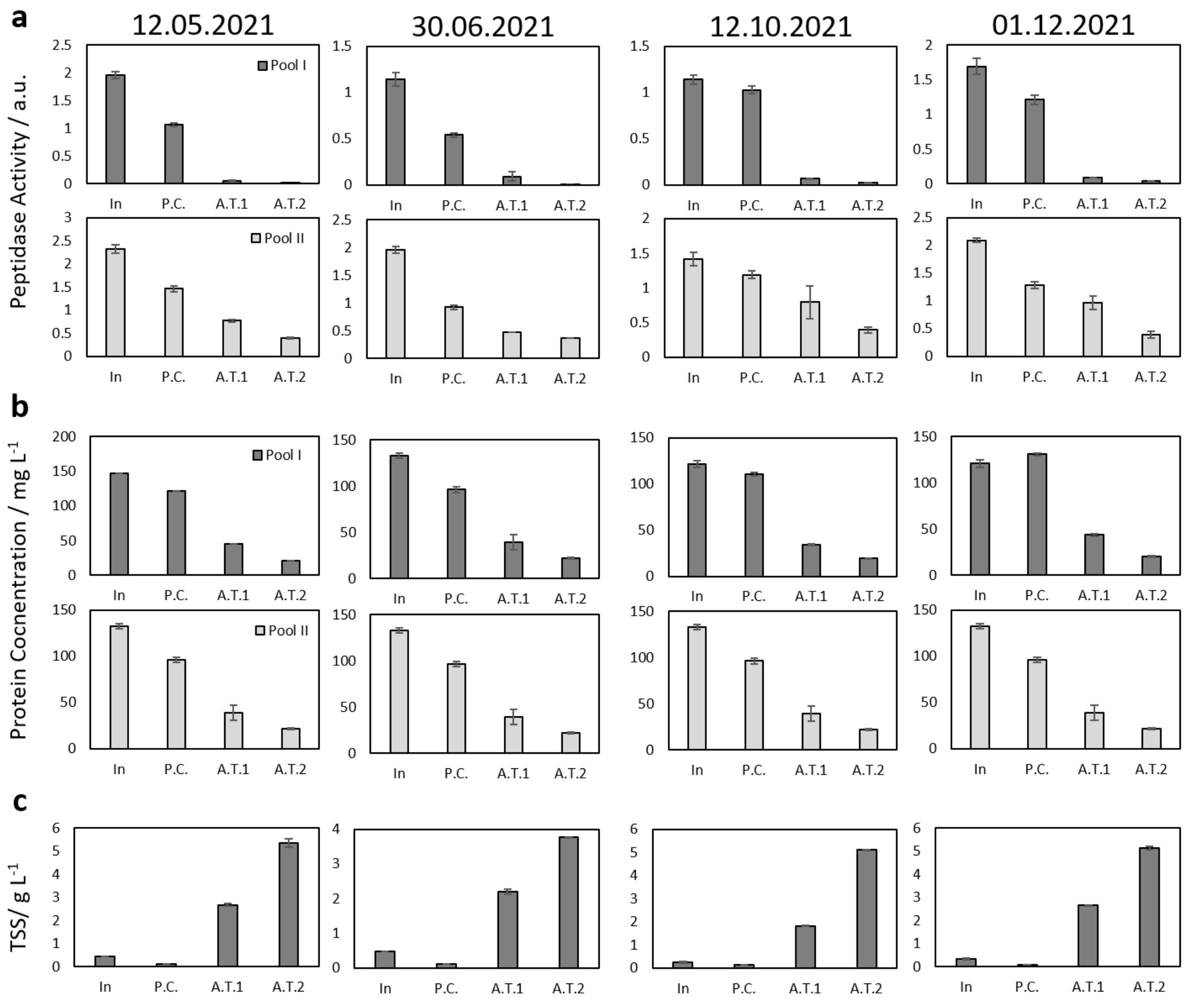

Figure S7. Seasonality of peptidase activity (a) and protein concentration (b) of protein extracts and total suspended solid contents (TSS, c) of untreated wastewater samples. Pool I: Dissolved extracellular protein; Pool II: Extrapolymeric substance (EPS) -bound and

dissolved extracellular protein. Samples were obtained from different stages of wastewater treatment plant A at four different time points throughout the year (In – influent, P.C. – primary clarification, A.T.1 – high-load aeration tank, A.T.2 – low-load aeration tank). Data points and error bars represent means +/- standard deviations of triplicate extractions.

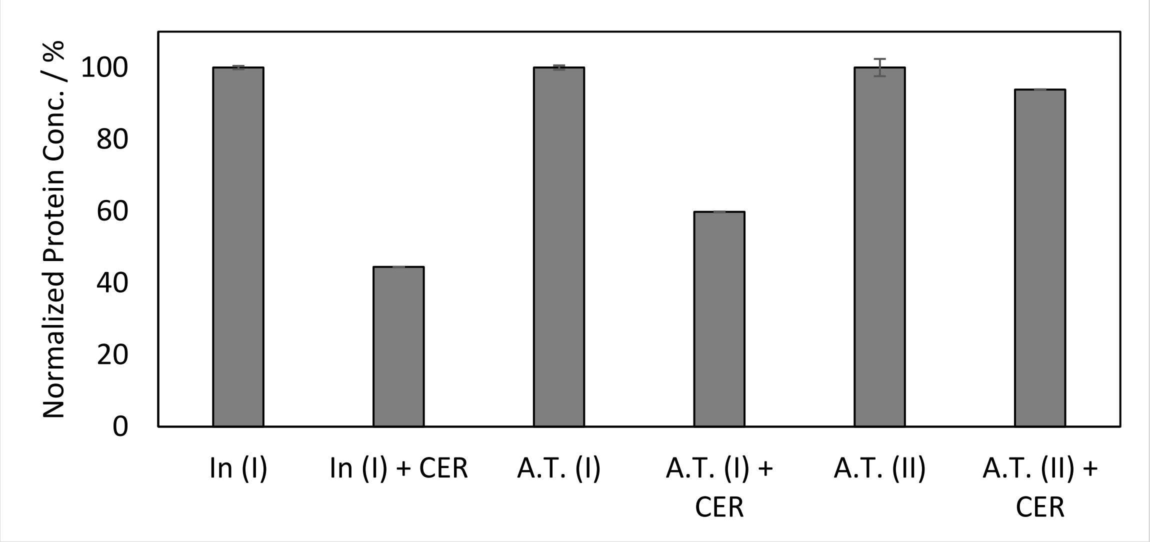

Figure S8. Sorption of protein to the cation exchange resin (CER) used for the disruption of extra polymeric substance (EPS). Shown are normalized protein concentrations of WWTP extracts obtained from WWTP A. In (I): Pool I influent extracts; In (I) + CER: Pool I influent extracts incubated with CER for 30 minutes; A.T. (I): Pool I extracts from high-load aeration tank; A.T. (I) + CER: Pool I extracts from high-load aeration tank incubated with CER for 30 minutes. A.T. (II): Pool II extracts from high-load aeration tank; A.T. (II) + CER: Pool II extracts from high-load aeration tank incubated with CER for 30 minutes. Normalization to respective reference sample. Data points and error bars represent normalized means +/- standard deviation of triplicate experiments.

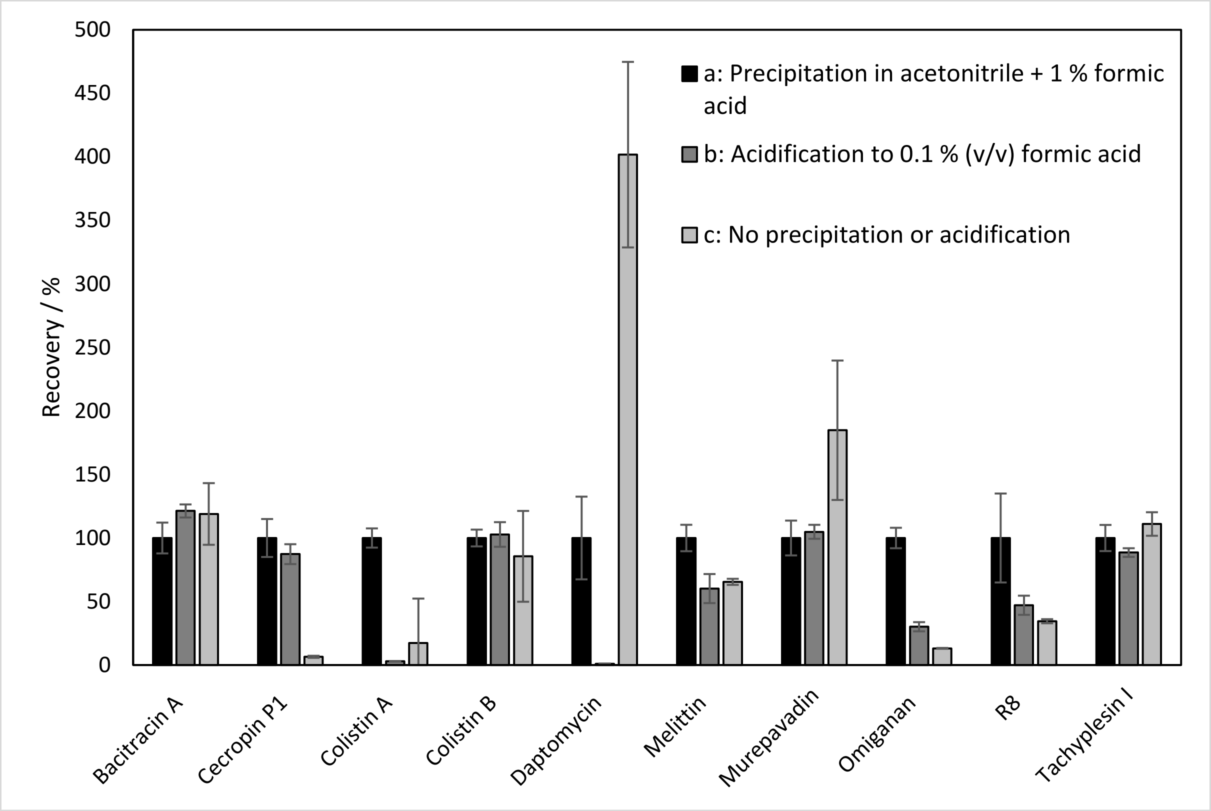

Figure S8. Control experiments stopping enzymatic processes to optimize the antimicrobial peptide (AMP) recovery. a: Standard protocol including precipitation of proteins in acetonitrile + 1 % formic acid (FA). b: No precipitation, sample acidified with 0.1 % FA. c: No precipitation or acidification. Data points and error bars represent mean +/- standard deviation of triplicate incubations. All data points are normalized to protocol “a” for each AMP.

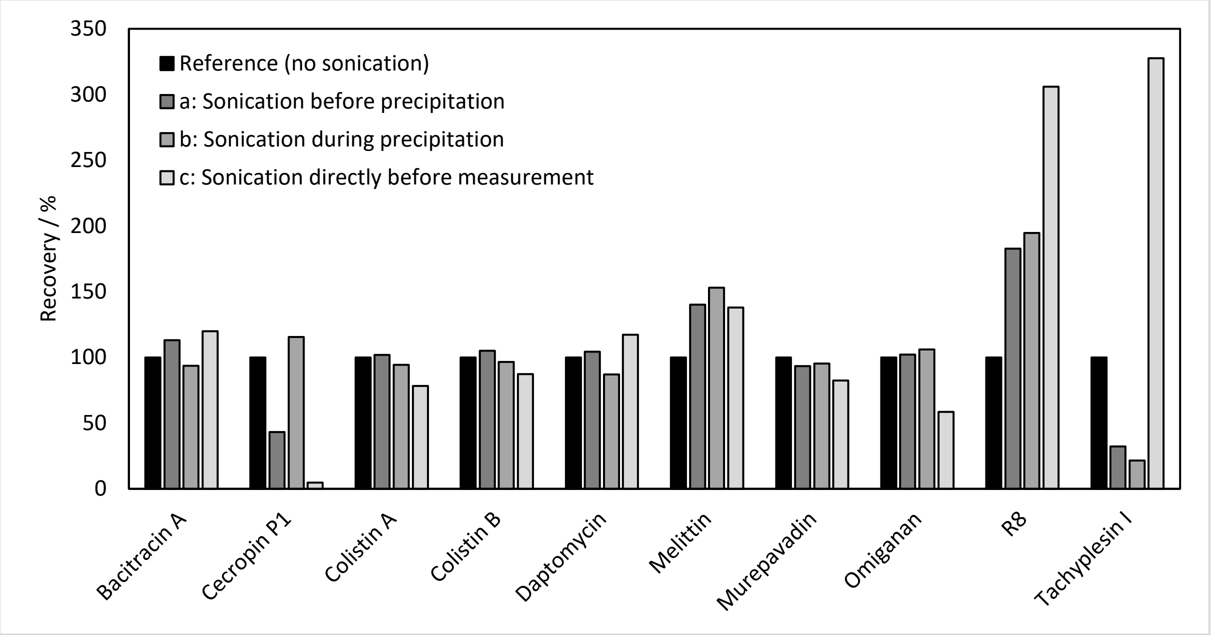

Figure S10. Effect of sonication during sample preparation on antimicrobial peptide (AMP) recovery. Reference refers to standard protocol without sonication. a: Sonication (5 min) before precipitation. b: Sonication (5 min) during precipitation. c: Sonication (5 min) after sample preparation, right before analysis. Data points represent single measurements.

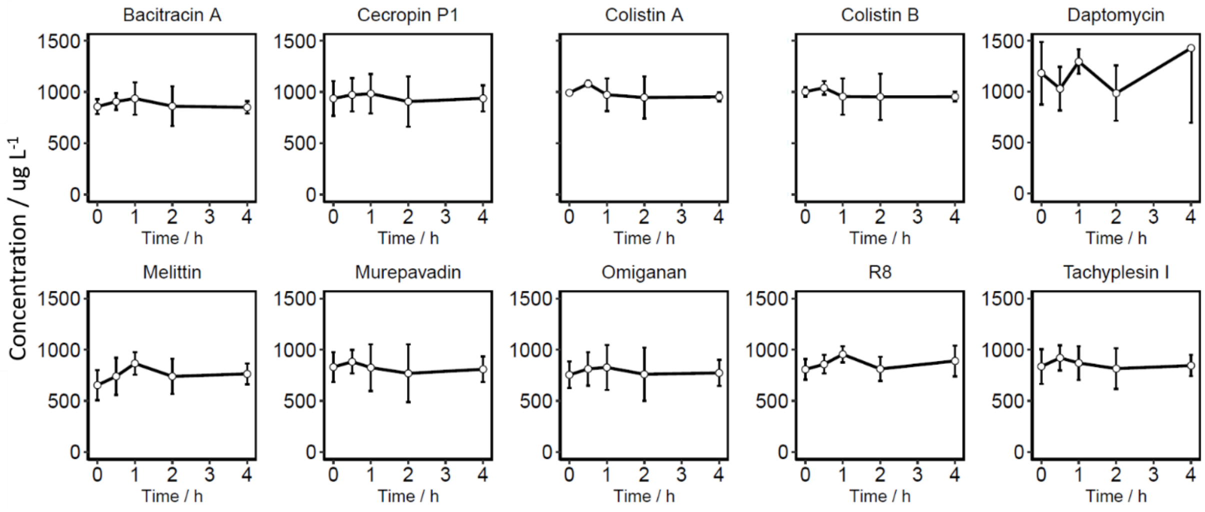

Figure S11. Stability of antimicrobial peptides (AMPs) in ultrapure water. Data points and error bars represent mean +/- standard deviation of four incubation experiments. For Colistin, the sum of Colistin A and B was 1000 µg / L, with an unknown A/B ratio. Therefore, no y-axis label is provided for these two AMPs.

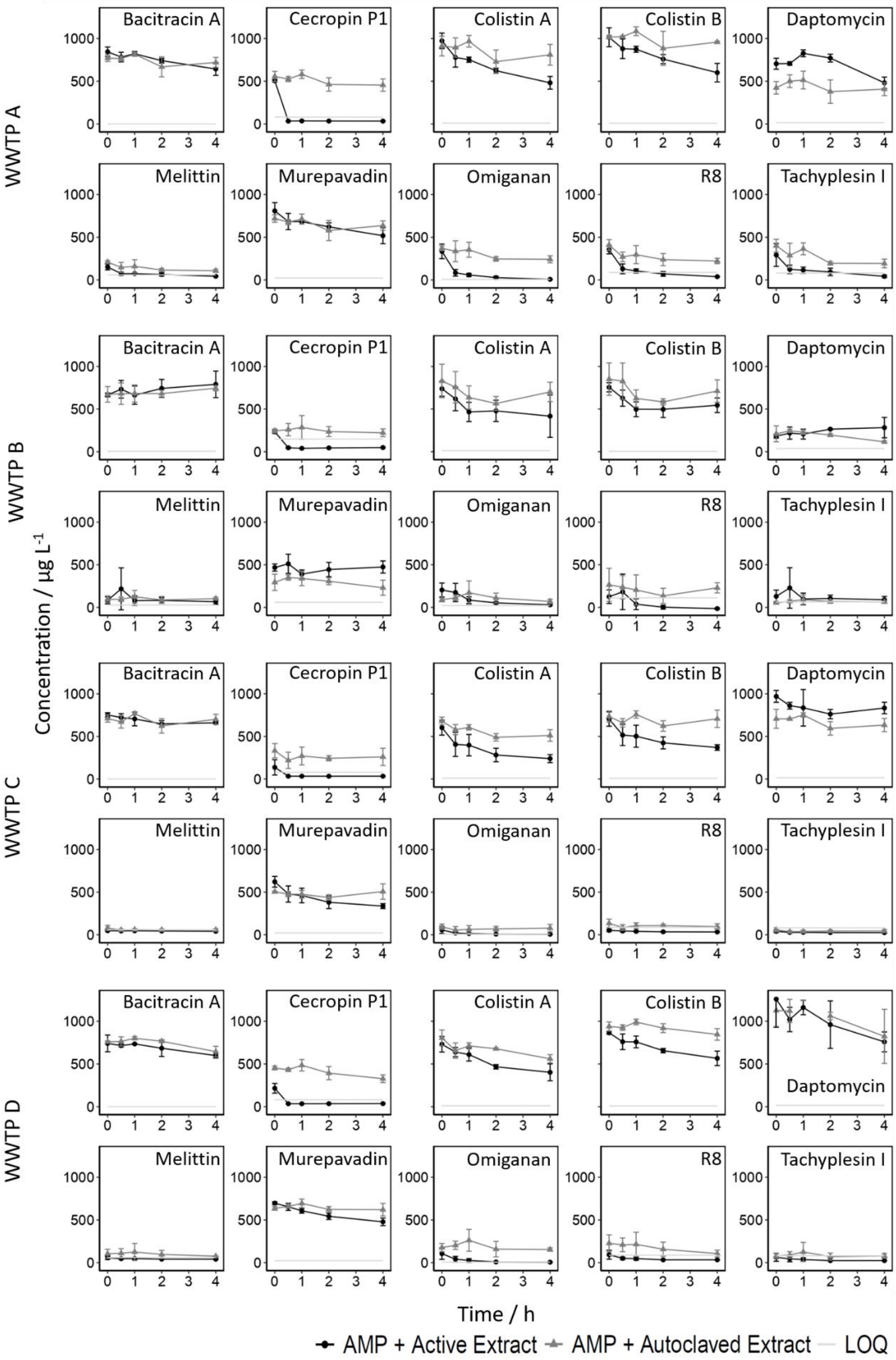

Figure S12. Progress curves of antimicrobial peptide (AMP) incubation with wastewater extracts from four full-scale wastewater treatment plants (WWTPs). Data points and error bars represent means +/- standard deviations of triplicate incubations. For Colistin, the sum of Colistin A and B was 1000 µg / L, with an unknown A/B ratio. Therefore, no y-axis label is provided for these two AMPs.

Table S6. Overview of transformation products (TPs) for Cecropin P1. Shown are chemical formulas of TPs, TP identifier indicated in Roman numerals, exact and measured mass to charge ratios (m/z), the deviation of exact and measured mass to charge ratios in ppm and the retention times (RT) of the TPs in minutes.

AMP	Chemical Formula TP	Exact m/z	Measured m/z	Mass deviation / ppm	RT / min
Cecropin P1	(IV) $C_{20}H_{28}N_4O_5$	405.2132	405.2133	0.25	7.70
Cecropin P1	(I) $C_{29}H_{45}N_7O_8$	310.6738	310.6738	0	6.12
Cecropin P1	(III) $C_{26}H_{46}N_{10}O_8$	314.1823	314.1823	0	1.47
Cecropin P1	(II) $C_{23}H_{33}N_5O_7$	492.2453	492.2454	0.20	7.36
Cecropin P1	(V) $C_{29}H_{51}N_{11}O_9$	349.7008	349.7008	0	1.97
Cecropin P1	(XII) $C_{60}H_{104}N_{18}O_{19}$	691.3935	691.3930	-0.72	7.37
Cecropin P1	(VI) $C_{36}H_{57}N_9O_{11}$	396.7162	396.7162	0	6.33
Cecropin P1	(VII) $C_{51}H_{88}N_{16}O_{16}$	591.3355	591.3355	0	7.12
Cecropin P1	(VIII) $C_{75}H_{127}N_{21}O_{23}$	423.4926	423.4925	-0.24	6.3
Cecropin P1	(X) $C_{33}H_{52}N_8O_{10}$	361.1976	361.1974	-0.55	6.27
Cecropin P1	(IX) $C_{54}H_{92}N_{14}O_{14}$	387.9045	387.9042	-0.77	5.95
Cecropin P1	(XI) $C_{38}H_{67}N_{13}O_{11}$	441.7614	441.7614	0	5.93
Cecropin P1	(XIII) $C_{42}H_{69}N_{14}O_{14}$	307.5115	307.5114	-0.33	5.42

Table S7. Validation of transformation products (TPs) of Cecropin P1 by MS2 data. Shown are chemical formulas of TPs, MS2 fragments in Roepstorff-Fohlmann-Biemann format [1,2], exact and measured mass to charge ratios of each fragment and the deviations of the exact and measured mass to charge ratios in ppm.

Transformation product	Fragment	Exact m/z	Measured m/z	Deviation / ppm
$C_{20}H_{28}N_4O_5$	W b2	274.1186	274.1182	-1.46
	W z2	301.1547	301.1547	0
	W a2	246.1237	246.1234	-1.22
$C_{29}H_{45}N_7O_8$	L y3	347.2289	347.2285	-1.15
	W b2	274.1186	274.1182	-1.46
	S y2	234.1448	234.1446	-0.85
$C_{26}H_{46}N_{10}O_8$	Q y5	514.2732	514.2733	0.19
	G y4	386.2146	386.2142	-1.04
	Q b2	242.1499	242.1498	-0.41
$C_{23}H_{33}N_5O_7$	L y2	219.1339	219.1337	-0.91
	W b2	274.1186	274.1182	-1.46
	L b3	387.2027	387.2028	0.26
$C_{29}H_{51}N_{11}O_9$	Q y5	514.2732	514.2741	1.75
	G y4	386.2146	386.2137	-2.33
	Q b3	313.1870	313.1870	0
	I b2	185.1285	185.1282	-1.62
$C_{60}H_{104}N_{18}O_{19}$	S y 13	1268.6957	1268.6949	-0.63

	A y7	698.3944	698.3944	0
	Q y5	514.2732	514.2720	-2.33
$C_{36}H_{57}N_9O_{11}$	L y5	519.3137	519.3134	-0.58
	W b2	274.1186	274.1180	-2.19
	L b3	387.2027	387.2030	0.77
$C_{51}H_{88}N_{16}O_{16}$	A y9	882.5156	882.5138	-2.04
	A y7	698.3944	698.3936	-1.15
	I y6	627.3573	627.3557	-2.55
	A b6	555.3137	555.3149	2.16
$C_{75}H_{127}N_{21}O_{23}$	K y11	609.3642	609.3626	-2.63
	L y13	473.2840	473.2834	-1.27
	K y7	395.2269	395.2270	0.25
$C_{33}H_{52}N_8O_{10}$	L y4	448.2766	448.2752	-3.12
	W y5	317.6816	317.6808	-2.52
	K y2	248.1605	248.1593	-4.89
	W a2	246.1237	246.1233	-1.63
$C_{54}H_{92}N_{14}O_{14}$	L y8	444.7975	444.7968	-1.57
	W b2	274.1186	274.1181	-1.82
	W y9	537.8371	537.8348	-4.28
$C_{38}H_{67}N_{13}O_{11}$	A y7	698.3944	698.3962	2.58
	Q y5	514.2732	514.2729	-0.58
	I y6	314.1823	314.1815	-2.55
$C_{42}H_{69}N_{14}O_{14}$	S y5	534.3246	534.3247	0.19
	K y4	447.2926	447.2933	1.56
	L y6	324.2080	324.2076	-1.23

Table S8. Overview of transformation products (TPs) for R8. Shown are chemical formulas of TPs, TP identifier in Roman numerals, exact and measured mass to charge ratios (m/z), the deviation of exact and measured mass to charge ratios in ppm and the retention times (RT) of the TPs in minutes.

AMP	Chemical Formula of TP	Exact m/z	Measured m/z	Deviation / ppm,	RT / min
R8	(IV) $C_{17}H_{25}N_3O_4$	336.1918	336.1914	-1.19	6.30
R8	(VII) $C_{27}H_{44}N_6O_6$	275.1734	275.1733	-0.36	5.69
R8	(II) $C_{28}H_{46}N_6O_6$	282.1812	282.1810	-0.71	6.14
R8	(V) $C_{30}H_{49}N_7O_7$	310.6919	310.6915	-1.29	6.19
R8	(I) $C_{37}H_{55}N_7O_7$	355.7154	355.7152	-0.56	7.44
R8	(VI) $C_{50}H_{79}N_{11}O_{10}$	497.8078	497.8085	1.41	6.70
R8	(III) $C_{52}H_{83}N_{11}O_{10}$	341.5514	341.5515	0.29	7.26
R8	(VIII) $C_{49}H_{78}N_{10}O_9$	317.8724	317.8723	-0.31	7.29
R8	(IX) $C_{36}H_{61}N_9O_8$	374.7394	374.7397	0.80	5.23
R8	(X) $C_{43}H_{67}N_9O_8$	280.1777	280.1778	0.36	6.30
R8	(XI) $C_{23}H_{37}N_5O_5$	232.6470	232.6470	0.00	5.13
R8	(II) $C_{22}H_{35}N_5O_5$	225.6392	225.6392	0.00	3.25
R8	(XII) $C_{100}H_{156}N_{22}O_{20}$	497.3039	497.3039	0.00	9.47

Table S9. Validation of transformation products (TPs) of R8 by MS2 data. Shown are chemical formulas of TPs, MS2 fragments in Roepstorff-Fohlmann-Biemann format[1,2], exact and measured mass to charge ratios of each fragment and the deviations of the exact and measured mass to charge ratios in ppm.

Transformation product	Fragment	Exact m/z	Measured m/z	Deviation / ppm
$C_{17}H_{25}N_3O_4$	F a1	120.0808	120.0809	0.83
	L a2	233.1648	233.1647	-0.43
	L b2	261.1598	261.1596	-0.77
$C_{27}H_{44}N_6O_6$	F y4	450.2711	450.2715	0.89
	G y3	303.2027	303.2021	-1.98
	K y2	246.1812	246.1816	1.62
$C_{28}H_{46}N_6O_6$	L y4	416.2867	416.2858	-2.16
	G y3	303.2027	303.2024	-0.99
	L a2	233.1648	233.1646	-0.86
$C_{30}H_{49}N_7O_7$	G y3	303.2027	303.2026	-0.33
	V a2	143.1179	143.1179	0.00
	V b2	171.1128	171.1126	-1.17
$C_{37}H_{55}N_7O_7$	L y5	563.3552	563.3554	0.36
	G y4	450.2711	450.2709	-0.44
	L a2	233.1648	233.1646	-0.86
$C_{50}H_{79}N_{11}O_{10}$	F y8	448.2736	448.2716	-4.46
	G y3	303.2027	303.2027	0.00
	K y7	374.7394	374.7385	-2.40

$C_{52}H_{83}N_{11}O_{10}$	L y8	438.2893	438.2889	-0.91
	G y7	381.7473	381.7476	0.79
	L a2	233.1648	233.1647	-0.43
$C_{49}H_{78}N_{10}O_{9}$	L y7	402.7707	402.7706	-0.25
	G y6	346.2287	346.2281	-1.73
	V y4	506.3337	506.3338	0.20
$C_{36}H_{61}N_{9}O_{8}$	V y5	549.3395	549.3415	3.64
	G y3	303.2027	303.2017	-3.30
	V y1	118.0863	118.0867	3.39
	K b1	129.1022	129.1023	0.77
	A y6	620.3766	620.3773	1.13
$C_{43}H_{67}N_{9}O_{8}$	L y6	346.2287	346.2282	-1.44
	G y5	289.6867	289.6859	-2.76
	L a2	233.1648	233.1648	0.00
$C_{23}H_{37}N_{5}O_{5}$	L y3	317.2183	317.2190	2.21
	F a1	120.0808	120.0809	0.83
$C_{22}H_{35}N_{5}O_{5}$	G y3	303.2027	303.2025	-0.66
	F a1	120.0808	120.0809	0.83
$C_{100}H_{156}N_{22}O_{20}$				

Table S10. Overview of transformation products (TPs) for Omiganan. Shown are chemical formulas of TPs, TP identifiers in Roman numerals, exact and measured mass to charge ratios (m/z), the deviation of exact and measured mass to charge ratios in ppm and the retention times (RT) of the TPs in minutes.

AMP	Chemical Formula TP	Exact m/z	Measured m/z	Deviation / ppm	RT / min
Omiganan	(II) $C_{72}H_{90}N_{16}O_{10}$	670.3586	670.3585	-0.15	10.30
Omiganan	(IV) $C_{84}H_{114}N_{24}O_{12}$	413.7335	413.7331	-0.97	8.38
Omiganan	(I) $C_{61}H_{80}N_{14}O_9$	577.3189	577.3190	0.17	9.54
Omiganan	(IX) $C_{18}H_{36}N_6O_4$	401.2871	401.2872	0.25	13.74
Omiganan	(III) $C_{78}H_{102}N_{20}O_{11}$	499.2752	499.2753	0.20	9.18
Omiganan	(VI) $C_{34}H_{53}N_9O_6$	342.7132	342.7131	-0.29	7.28
Omiganan	(V) $C_{72}H_{93}N_{21}O_9$	349.9439	349.9437	-0.57	7.34
Omiganan	(VIII) $C_{45}H_{63}N_{11}O_7$	435.7529	435.7532	0.69	8.52
Omiganan	(VII) $C_{18}H_{39}N_{11}O_3$	229.6691	229.6691	0.00	0.75

Table S11. Validation of transformation products (TPs) of Omiganan by MS2 data. Shown are chemical formulas of TPs, MS2 fragments in Roepstorff-Fohlmann-Biemann format[1,2], exact and measured mass to charge ratios of each fragment and the deviations of the exact and measured mass to charge ratios in ppm.

Transformation product	Fragment	Exact m/z	Measured mass	Mass deviation / ppm
$C_{72}H_{90}N_{16}O_{10}$	P y2	302.1499	302.1495	-1.32
	W b7	1038.5672	1038.5682	0.96
$C_{61}H_{80}N_{14}O_9$	W b7	519.7872	519.7859	-2.50
	R b3	383.2765	383.2775	2.61
$C_{78}H_{102}N_{20}O_{11}$	P y3	458.2510	458.2517	1.53
	W b7	519.7872	519.7897	4.81
	P b5	333.7079	333.7079	0.00
$C_{18}H_{36}N_6O_4$				
$C_{84}H_{114}N_{24}O_{12}$				
$C_{34}H_{53}N_9O_6$				
$C_{72}H_{93}N_{21}O_9$				
$C_{45}H_{63}N_{11}O_7$				
$C_{18}H_{39}N_{11}O_3$				

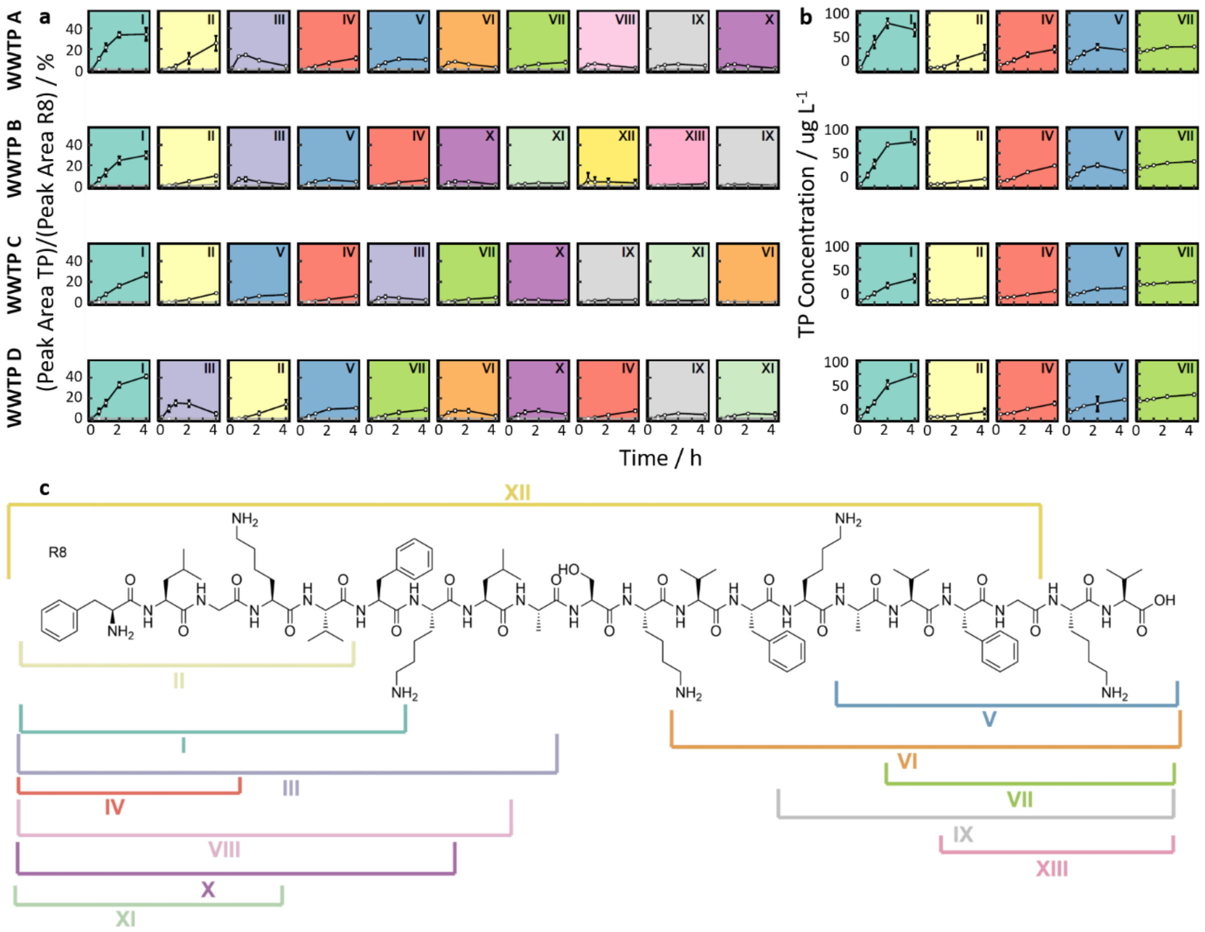

Figure S13. Transformation products (TPs) of antimicrobial peptide R8. **a:** Progress curves of the ten most abundant TPs of single hydrolytic events of R8 relative to the peak area of the calibration of R8 at 1 mg/L. R8 was incubated with dissolved extracellular enzymes from the influents of wastewater treatment plants (WWTPs) A-D. Same colors indicate same molecules. **b:** Absolute quantification of selected custom-synthesized TPs. **c:** Chemical structure of R8 and the TPs depicted in a and b. Data points and error bars represent means +/- standard deviations of triplicate enzyme extractions.

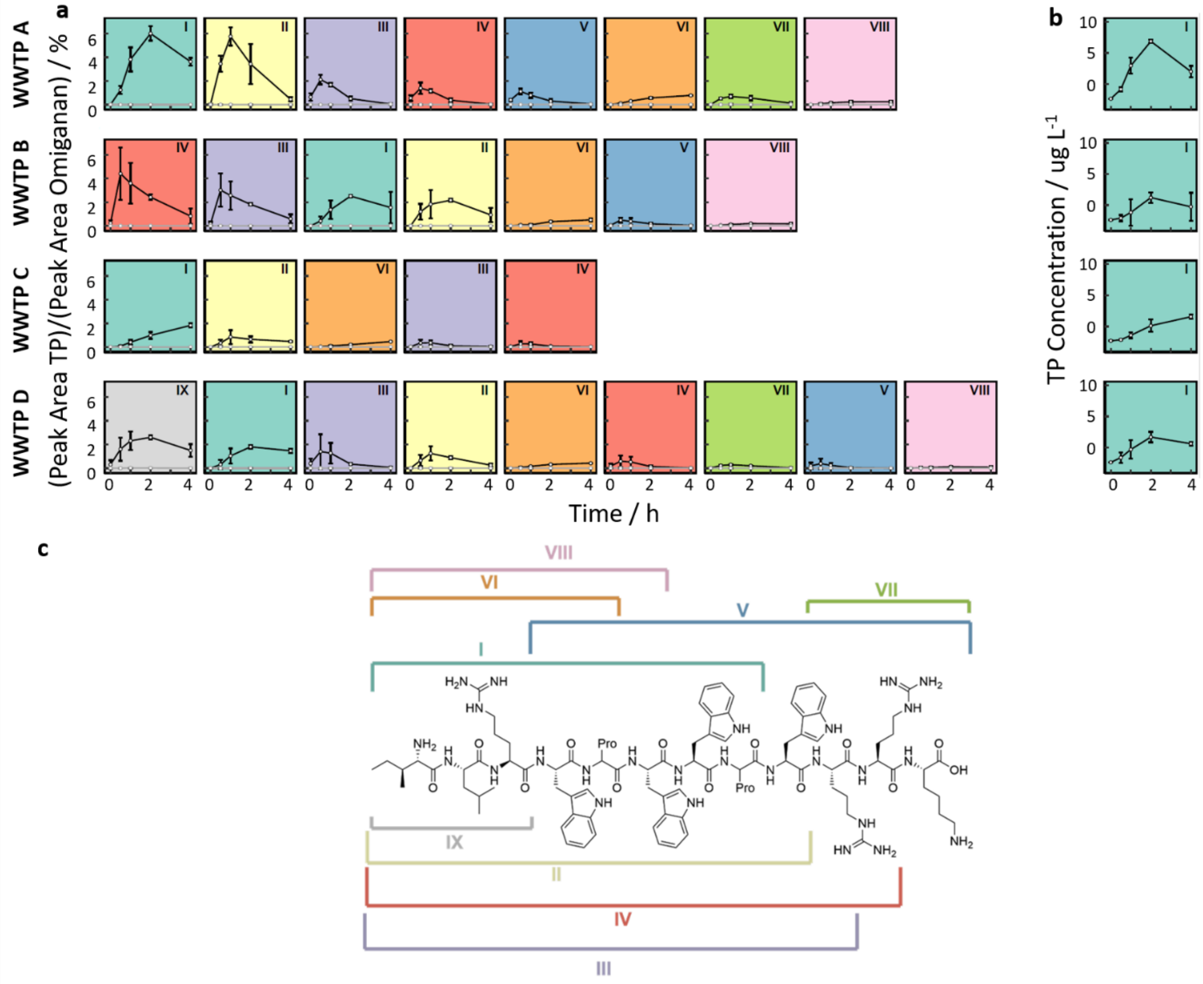

Figure S14. Transformation products (TPs) of antimicrobial peptide Omiganan. **a:** Progress curves of the ten (if available) most abundant TPs of single hydrolytic events of Omiganan relative to the peak area of the calibration of Omiganan at 1 mg/L. Omiganan was incubated with dissolved extracellular enzymes from the influents of wastewater treatment plants (WWTPs) A-D. Same colours indicate same molecules. **b:** Absolute quantification of selected custom-synthesized TPs. **c:** Chemical structure of Omiganan and the TPs depicted in a and b. Data points and error bars represent means +/- standard deviations of triplicate enzyme extractions.

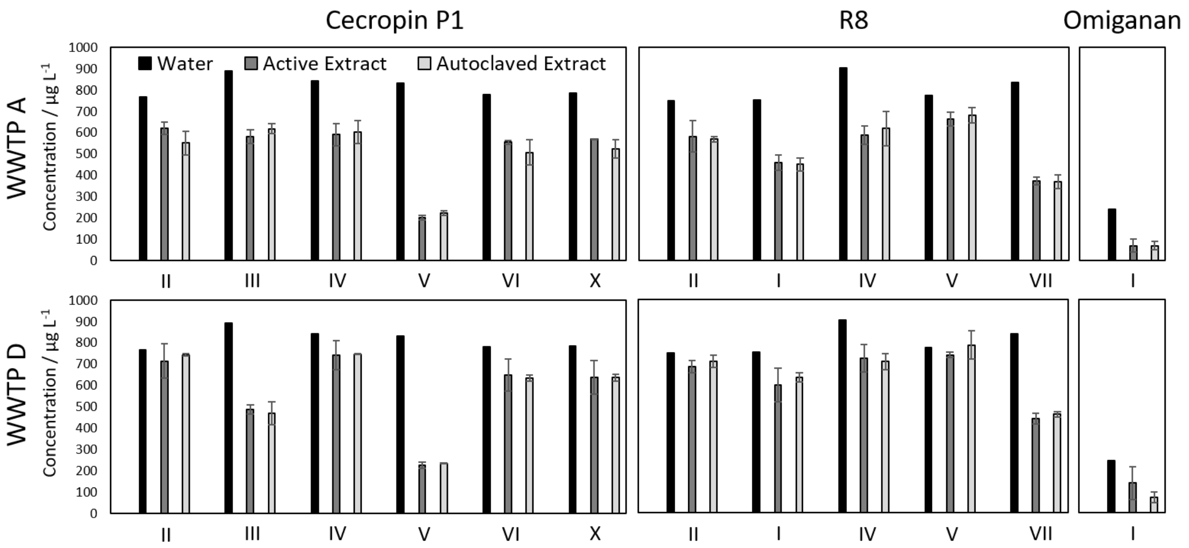

Figure S15. Incubation of custom-synthesized transformation products (TPs) with ultrapure water, as well as active and autoclaved wastewater extracts from wastewater treatment plants (WWTPs) A and D. Recovery was determined directly after spiking the TPs to the respective medium. TPs are numbered according to Figure 3, S12, and S13. Data points and error bars represent means +/- standard deviations of triplicate incubations (for ultrapure water incubations, single incubations were performed).

Table S12. Sorption potential of the custom synthesized transformation products (TPs) to the wastewater matrix in wastewater treatment plants (WWTPs) A and D. Sorption was calculated from difference of TP concentration in spiked ultrapure water and TP concentration in spiked autoclaved wastewater extract. TPs are numbered according to Figure 3, S12, and S13 and their peptide sequence is indicated. Mean values and standard deviations of triplicate incubations are provided. The maximum concentration of the respective TP during the incubation experiments of antimicrobial peptides with wastewater extracts is provided, as well as the potential concentration when accounting for the sorption processes of the wastewater matrix.

Cecropin P1	Sorption in WWTP A extracts (in %)	Standard deviation	Sorption in WWTP D extracts (in %)	Standard deviation	Maximum conc. detected in exp. (in ug/L)	Potential conc. considering sorption (in ug/L)
SWLS (II)	21.7	5.5	2.5	0.4	57.1	69.5
IQGGPR (III)	27.3	2.3	42.2	5.3	87.8	124.8
SWL (IV)	24.1	5.3	9.6	0.3	2.7	3.4
AIQGGPR (V)	61.0	1.0	59.8	0.2	48.6	78.2
SWLSKTA (VI)	27.3	5.8	14.6	1.5	28.6	36.4
SWLSKT (X)	26.1	4.2	14.8	1.6	2.9	3.7
R8						
FLGKV (II)	17.9	1.3	4.1	2.9	16.5	19.5
FLGKVF (I)	30.1	3.2	12.0	2.2	78.3	101.9
FLG (IV)	28.3	8.0	19.5	3.8	22.8	29.3
AVFGKV (V)	9.2	3.7	-1.2	6.6	27.5	30.0
VFGKV (VII)	46.7	3.3	37.7	1.2	32.1	47.1
Omiganan						
ILRWPWWP (I)	17.2	2.1	17.0	2.6	6.9	8.1

(1) Roepstorff, P.; Fohlman, J. Letter to the Editors. *Biol. Mass Spectrom.* **1984**, *11* (11), 601–601. https://doi.org/10.1002/bms.1200111109.

(2) Johnson, R. S.; Martin, S. A.; Biemann, K.; Stults, J. T.; Watson, J. T. Novel Fragmentation Process of Peptides by Collision-Induced Decomposition in a Tandem Mass Spectrometer: Differentiation of Leucine and Isoleucine. *Anal. Chem.* **1987**, *59* (21), 2621–2625. https://doi.org/10.1021/ac00148a019.
